# Investigating the effect of lifestyle risk factors upon number of aspirated and mature oocytes in in vitro fertilization cycles: Interaction with antral follicle count

**DOI:** 10.1371/journal.pone.0221015

**Published:** 2019-08-16

**Authors:** Lana Salih Joelsson, Evangelia Elenis, Kjell Wanggren, Anna Berglund, Anastasia N. Iliadou, Carolyn E. Cesta, Sunni L. Mumford, Richard White, Tanja Tydén, Alkistis Skalkidou

**Affiliations:** 1 Institute of Women’s and Children’s, Obstetrics and Gynecology, Uppsala University, Uppsala, Sweden; 2 Department of Clinical Science, Intervention and Technology, Obstetrics and gynecology, Karolinska Institute, Stockholm, Sweden; 3 National Centre for Knowledge on Men's Violence against women (NCK), Uppsala University, Uppsala, Sweden; 4 Department of Medical Epidemiology and Biostatistics, Karolinska Institute, Stockholm, Sweden; 5 Epidemiology Branch, Division of Intramural Population Health Research, *Eunice Kennedy Shriver* National Institute of Child Health and Human Development, National Institutes of Health, Bethesda, Maryland, United States of America; 6 Norwegian Institute of Public Health, Oslo, Norway; University of Porto, PORTUGAL

## Abstract

**Introduction:**

There is evidence demonstrating that certain lifestyle factors have a detrimental effect on fertility. Since such factors often coexist, possible synergistic effects merit further investigation. Thus we aimed to examine the cumulative impact of lifestyle factors on in vitro fertilization (IVF) early reproductive treatment outcomes and their interaction with measures of ovarian reserve.

**Materials and methods:**

By following women who were starting their first fresh IVF cycle in 2 cohorts, the “Lifestyle study cohort” (hypothesis generating cohort, n = 242) and the “UppSTART study” (validation cohort, n = 432) in Sweden, we identified two significant risk factors acting independently, smoking and BMI, and then further assessed their cumulative effects.

**Results:**

Women with both these risk factors had an Incidence Rate Ratio (IRR) of 0.75 [(95% CI 0.61–0.94)] regarding the number of aspirated oocytes compared to women without these risk factors. Concerning the proportion of mature oocytes in relation to the total number of aspirated oocytes, the interaction between BMI and Antral Follicle Count (AFC) was significant (p-value 0.045): the lower the value of AFC, the more harmful the effect of BMI with the outcome.

**Conclusions:**

Data shows that there is an individual as well as a cumulative effect of smoking and BMI on the number of aspirated and mature oocytes in fresh IVF treatment cycles. AFC might modify associations between BMI and the proportion of mature oocytes in relation to the total number of aspirated oocytes. These results highlight the importance of lifestyle factors on IVF early reproductive outcomes and provide additional evidence for the importance of preconception guidance for the optimization of IVF cycle outcome.

## Introduction

Infertility is a serious concern for individuals wishing to start a family and can be costly for both the couple and for society. Approximately 10–15% of those who try to conceive suffer from infertility and often turn to medically assisted reproduction techniques [1]. Contrary to the perception of many, undergoing In Vitro Fertilization (IVF) does not guarantee success; 38–69% of couples who begin IVF will remain childless, even if they undergo as many as six IVF cycles [2]. This underlies the importance of identification of potentially modifiable predictors of successful treatment. Lifestyle factors including obesity, smoking, stress, and most importantly, postponing parenthood until an advanced age, have been shown to contribute to reduced fertility [3].

The relevance of personal behavior and lifestyle-related factors that may adversely affect fertility and ART outcomes has increasingly been discussed over the last ten years [4,5]. Reviewing the impact of certain lifestyle habits on IVF outcomes can motivate patients to modify behaviors such as smoking, heavy alcohol consumption or excessive body weight, since changes may actually improve a couple’s chance of conceiving through IVF.

While there is increasing evidence demonstrating that lifestyle factors have deteriorating effects on fertility, little is known about the exact pathway mediating their effect on the success of ART [6–8]. Furthermore, few studies have specifically evaluated the combined effect of different lifestyle factors, including obesity, smoking or alcohol drinking, on early fertility treatment outcomes, such as quantity and quality of retrieved oocytes [9,10] which in turn is a robust surrogate marker for clinical success of IVF treatment [11,12]. Consequently, since negative lifestyle factors often co-occur, it cannot be ruled out that there is a synergistic effect, which in turn warrants further research. Hence, the primary aim of the present study was to elucidate the influence of individual, as well as cumulative effect of lifestyle factors on early reproductive outcomes after a first completed IVF cycle. A secondary aim was to also investigate the possible interaction of lifestyle factors with antral follicle count. By employing two different prospective cohorts, we were able to use one cohort to generate hypotheses (identify potential risk factors) and the other cohort to validate them.

## Materials and methods

### Design

This was a cohort study based on 2 different prospective cohorts.

### Participants and recruitment

#### “Lifestyle study cohort” (hypothesis-generating cohort)

The “Lifestyle study cohort” consists of women attending eight fertility clinics in Sweden (six public and two private). Consecutive women (n = 874) were asked to participate in the study at their first visit to the clinic. The time of this first visit to the clinic was defined as ‘baseline year’. Those who agreed to participate received a questionnaire (S1 and S2 Questionnaire) to complete either at the clinic or at home (returning it by mail in a prepaid envelope). The completed questionnaires were returned including the signed informed consent. Data collection started in May 2013 and ended in September 2015. Further details on this cohort can be found in a previous publication [13].

Of 874 eligible women, 747 (85.4%) agreed to participate and 466 (62.3%) completed the questionnaire. Follow-up data regarding the reproductive outcomes were obtained from the medical records of 442 women in January 2017. Only women who completed their first IVF treatment (n = 242) were included in the study. Women undergoing donor oocyte, donor sperm and preimplantation genetic diagnosis (PGD) cycles were excluded.

The patients underwent IVF according to standard stimulation protocols. Women in the study cohort underwent ovarian stimulation with the use of a long GnRH agonist protocol in 31.0% of treatments and a GnRH antagonist protocol in 69.0%, with recombinant FSH in 78.5% of treatments and hMG in 21.5%. Ovulation was triggered mostly with the use of subcutaneous hCG, while the use of GnRH agonist was limited only among cases at risk of ovarian hyperstimulation syndrome (OHSS). Embryologists have determined the total number of oocytes retrieved per cycle and evaluated the maturity of the oocytes. Only metaphase II oocytes (MII) were considered mature. Normal fertilization, indicated by the presence of two clearly distinct pronuclei and the embryo quality was assessed on the day of transfer. The cleavage stage embryos were scored based on cell number and the degree of fragmentation, according to the grading system of Istanbul Consensus Workshop on Embryo Assessment [14]. The Regional Ethical Review Board in Uppsala approved of the study (2012/278).

#### “UppStART study” cohort (validation cohort)

The validation cohort was derived from the Uppsala-Stockholm Assisted Reproductive Technology (UppStART) study [15,16]; i.e. a prospective cohort study of couples undergoing their first IVF treatment in the greater Stockholm region (Stockholm and Uppsala County) (n = 432). Participants were recruited from one public and two private fertility and reproductive health clinics in Stockholm and one private clinic in Uppsala County, which serves a large volume of patients from Stockholm. Signed consent forms were sent to the UppStART research nurse at Karolinska Institute (KI) who monitored recruitment and questionnaire responses.

The participants were asked to answer an extensive web-based questionnaire within a few days of their clinical visit and prior to starting their IVF treatment, which included questions on sociodemographic, anthropometric and lifestyle factors. Of the total number of women who gave consent to participate in UppStART, 14% did not respond to the baseline questionnaire. Recruitment took place from September 2011 to December 2013 and participants were followed up until December 2014 (n = 432).

The IVF treatment protocols used in this study did not substantially differ from the one described above for the “Lifestyle study cohort” participants.

The Regional Ethical Review Board in Stockholm approved of the study (2011/230-31/1, 2011/1427-32, 2012/131-32, 2012/792-32, 2013/1700-32).

**Fig 1:**
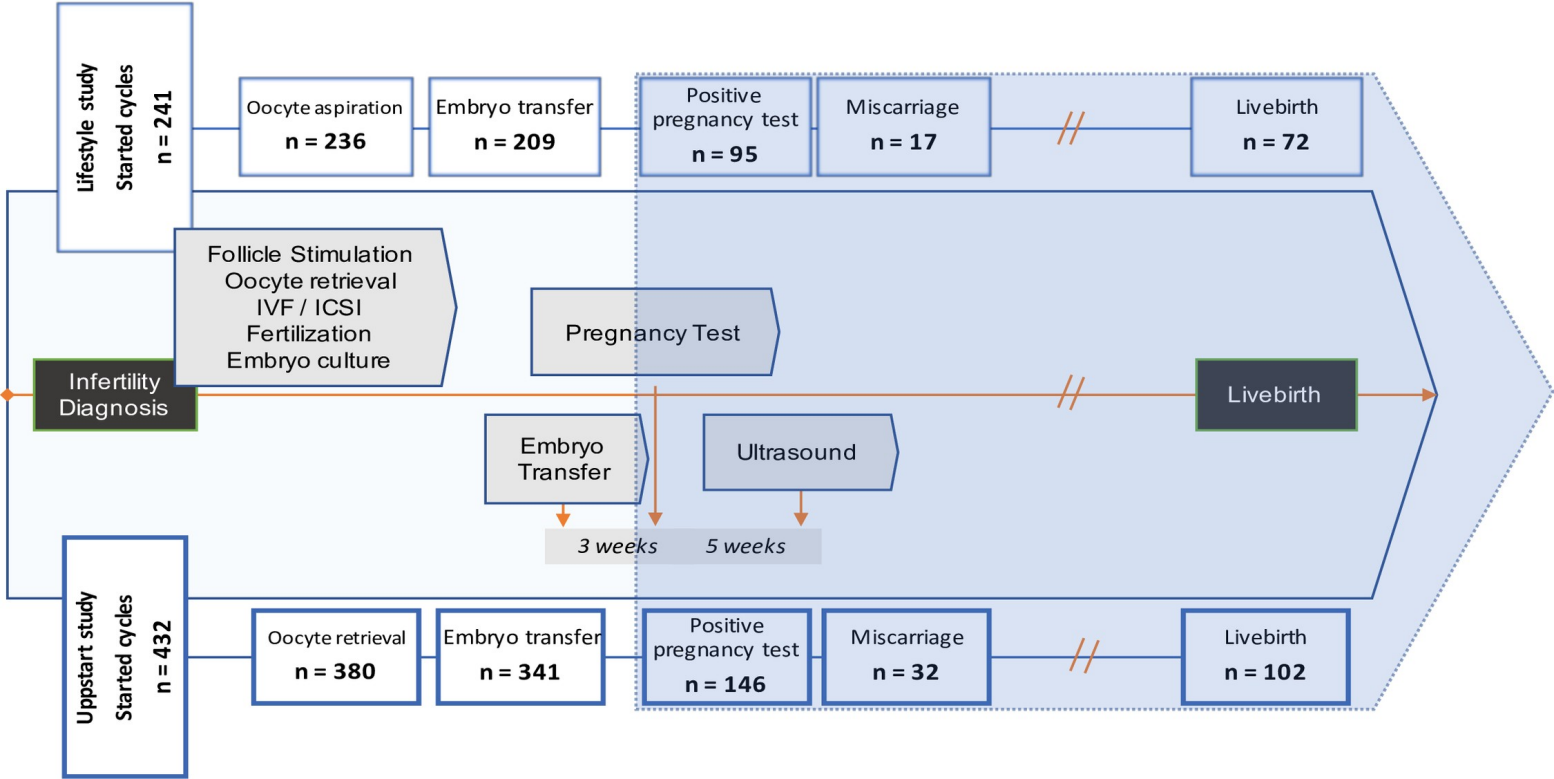
Flowchart of IVF cycles procedures included and their outcomes in the 2 study cohorts.

A flowchart describing participant progression through the study/IVF cycle is presented in Fig 1.

### Study variables

#### Background, risk lifestyle and infertility-related variables

Age at start of cycle (years), education level [divided into low (non-university) and high (university level education)], and body mass index (BMI) (kg/m^2^) (at the time of first visit to the fertility clinic) were assessed via medical records. Alcohol consumption was self-reported and defined as number of standard drinks per week. Questions in the “UppStART study cohort” were more detailed with regards to amount and type of alcohol. Smoking was defined as smoking currently or within the past year before their visit to the fertility clinic (yes/no). The daily intake of caffeine (mg) was estimated from the number of cups of coffee (77 mg/one cup of coffee or equivalent) in “UppStART study cohort”. In “Lifestyle study cohort” caffeine consumption in this study was assessed by asking women about their coffee consumption only. Physical activity level (PAL) was calculated using the validated ‘PAL’ method, with scores assigned according to different levels of reported occupational and leisure activity on the baseline questionnaire [17]. Cause of infertility was obtained from participants’ medical records at the start of the cycle and classified as male factor, female factor, and mixed/unknown causes. Duration of infertility was defined as the number of years trying to become pregnant. Antral follicle count (AFC) was defined as total antral follicles assessed by ultrasound examination. In this study, we used AMH and AFC as markers for ovarian reserve. Women in both study cohorts were tested for ovarian reserve using a combination of AFC and anti-Müllerian hormone (AMH) level at their first visit to the fertility clinic. Out of 242 women in the Lifestyle study 98.35% were tested for AFC and 82.24% were tested for AMH. Out of 432 women in the Upstart study 68.06% were tested for AFC and 79.87% were tested for AMH.

#### Predictor/exposure variables

We considered the following exposure variables as possible risk factors: age, BMI, smoking, alcohol consumption, daily caffeine consumption (only coffee) and physical activity score (PAL). In addition, the history of diagnosed depression (self-reported or via diagnosis registered in medical records) was also considered as one of the exposure variables.

#### Outcome variables

All outcome variables were derived from data originating from the participants’ medical records. The outcomes were total number of oocytes aspirated, number of mature oocytes, number of embryos created, as well as number of utilizable embryos (i.e. transferred fresh and frozen).

### Statistical analysis

All statistical analyses were performed using R 3.5.3. Data in the two cohorts were analyzed in an identical manner in order to determine if risk factors identified in the “Lifestyle study cohort” could be validated in the “UppStART study cohort” as having a cumulative additive effect. Baseline characteristics were presented as medians and interquartile range (IQR). To identify differences between the two cohorts, the non-parametric Mann Whitney test was used for continuous variables, while for the comparison of categorical variables, the Chi square test was conducted. For each of the outcomes and cohorts, a dataset consisting of the outcome and possible risk factors was created. Multiple imputations were used [18–20] in order to impute missing data and construct 20 imputed datasets for each outcome/cohort combination.

In the “Lifestyle study cohort”, a crude univariable quasi-poisson regression was performed evaluating the association between each of the outcomes in the study cohort and each of the risk factors. Additionally, identification of the effect modification of the risk factors by total AFC was attempted by using a likelihood ratio test. An adjusted multivariable analysis was then used, where all of the risk factors were included in a single model. Quasi-poisson regression was applied as the outcome variables were right-skewed. The effect estimates were pooled using standard statistical methodology for multiple imputation for nonresponse in surveys [21]. Variables that were significant at the alpha = 0,05 level in the “Lifestyle study cohort” in either the crude or adjusted analyses were considered to be potential risk factors for the respective outcomes.

We then wanted to see if we (based on our findings from the “Lifestyle study cohort”) could create a numerical risk score that could be validated in the “UppStART study”. The following was performed for each outcome: if two or more potential risk factors were found in the “Lifestyle study cohort”, we then proceeded to run the following analyses in the “UppStART study”. We began by creating a new variable “number of risk factors.” If the potential risk factor was dichotomous and showed an incidence rate ratio (IRR) less than one in the “Lifestyle study cohort”, then the variable "number of risk factors" was increased by one if the person had the potential risk factor (i.e. if a variable is expected to reduce the number of oocytes/embryos, that variable is a risk factor). Likewise, if the potential risk factor was dichotomous and showed an incidence rate ratio (IRR) greater than one in the “Lifestyle study” cohort, then the variable "number of risk factors" was increased by one if the person did not have the variable (i.e. if a variable is expected to increase the number of oocytes/embryos, it is a risk factor to not have that variable). If the risk factor was continuous, then it was dichotomized at the median (i.e. "greater than the median"/"lower than the median"). With regards to this model, we can interpret the coefficients as "percentage increase/decrease of outcome due to a one unit increase in the exposure". For example, we can take a model with an outcome of "mature oocytes " and an exposure of one risk factor vs a baseline of 0 risk factors. If the IRR = 1.04 it means that women with one risk factor are expected (on average) to have 4% more mature oocytes per oocytes aspirated than women with zero risk factors. If the IRR = 0.73 it means that women with one risk factor are expected (on average) to have 27% less mature oocytes per oocytes aspirated than women with zero risk factors.

For each outcome, we then ran four quasi-poisson statistical regression models: Model 1: No exposures; model 2: Model 1 + number of risk factors as a categorical exposure; model 3: Model 2 + Total AFC and model 4: model 3 + interaction between BMI and Total AFC.

The significance of number of risk factors was tested by using a likelihood ratio test to compare Models 1 and 2. Additionally, identification of the effect modification of the number of risk factors by total AFC was attempted by using a likelihood ratio test comparing models 3 and 4.

## Results

The participant´s background characteristics, lifestyle, and infertility related characteristics are summarized in Table 1. A total of 242 women who underwent their first fresh IVF cycle were evaluated in the “Lifestyle study cohort” and 432 women in the “UppStART study” validation cohort. The median age of the population at cycle start was lower in the “Lifestyle study cohort” [31.1 (IQR 6.8) years] compared to the validation cohort [34.0 (IQR 6.0) years]. There were more women with university education in the “UppStART study” validation cohort compared to the “Lifestyle study cohort”. The most common cause of infertility was unexplained/mixed and this was similar in both study populations. Furthermore, median AMH was higher in the “Lifestyle study” cohort whereas median AFC was lower in that group compared to the “UppStart study” cohort. The median number of oocytes retrieved was 9 (IQR 6.5) in the “Lifestyle study cohort” and 8 (IQR 6.0) in the “UppStART study” validation cohort (Table 1).

**Table 1:** Background characteristics, lifestyle variables and variables relating to infertility and infertility treatment among the participants of the two cohorts.

Characteristic	Lifestyle study N = 241		UppStART study N = 432		*P*-Value
Age (years), median (IQR)	31.1	(27.9–34.7)	34.0	(31.0–37.0)	<0.001
Level of education, n (%)					<0.001
College/university	153	(64.3)	334	(78.8)	
Non-college/non-university	85	(35.7)	90	(21.2)	
Body Mass Index (BMI) in kg/m^2^, median (IQR)	23.7	(21.4–27.9)	22.7	(20.9–25.6)	<0.001
Any alcohol consumption, *n* (%)	165	(69.0)	337	(78.0)	0.013
Smoking^a^, *n* (%)	78	(32.4)	148	(35.3)	0.493
Smoking^b^, *n* (%)	23	(9.5)	14	(3.3)	0.002
Caffeine- daily^c^ in mg/day, median (IQR)	221.1	(0.0–331.7)	221.1	(68.1–382.3)	<0.001
Physical activity score^d^, median (IQR)	1.7	(1.7–1.8)	1.7	(1.6–1.8)	0.064
History of depression, n (%)	42	(17.6)	58	(13.4)	0.183
Cause of infertility, n (%)					0.872
Mixed/Unknown	133	(55.2)	88	(54.0)	
Male	44	(18.3)	28	(17.2)	
Female	64	(26.6)	47	(28.8)	
Duration of infertility (years), median (IQR)	2.0	(1.5–3.0)	2.0	(2.0–3.0)	0.020
Total AFC, median (IQR)	10.0	(7.0–14.0)	16.0	(10.0–24.0)	<0.001
Total AFC< 7, N (%)	50	(21.0)	24	(8.2)	<0.001
AMH, median (IQR)	2.5	(1.6–4.7)	2.1	(1.1–3.8)	0.008
FSH- total dose for stimulation, median (IQR)	1513.5	(1198.5–2250.0)	1650	(1206.2–2610.0)	0.119
Number of aspirated oocytes, median (IQR)	9.0	(6.0–12.5)	8.0	(5.0–11.0)	0.003
Number of mature oocytes, median (IQR)	7.0	(5.0–11.0)	7.0	(4.0–11.0)	0.142
Number of embryos created, median (IQR)	4.0	(2.0–6.5)	4.0	(2.0–7.0)	0.621
Number of embryos used, median (IQR)	2.0	(1.0–4.0)	2.0	(1.0–4.0)	0.495

^a^ Smoking in general, currently or within the past year

^b^ currently smoking only.

^c^ 1 cup of coffee = 77mg caffeine

^d^ Physical activity score based on validated scale and reflecting different levels of occupational and leisure activity

### Effect of individual lifestyle risk factors

Women with high BMI had an adjusted IRR for the number of aspirated oocytes corresponding to 0.98 (95% CI: 0.97, 1.00) which means that with each unit increase in BMI, the women had 2% fewer aspirated oocytes (Table 2). There was also a significant decrease in number of created and utilizable embryos with increasing BMI, [IRR 0.97 (95% CI: 0.95, 0.99) and 0.96 (95% CI: 0.94, 0.99) respectively (S1 Table). Women who smoked had 19% fewer aspirated oocytes; however, this result was not significant in the “UppStART study” validation cohort (Table 2). Interestingly, there was a decrease in the number of created embryos and number of utilizable embryos in relation to alcohol intake in “UppStART study” validation cohort (S1 Table).

**Table 2:** Incidence rate ratio (IRR) and 95% Confidence intervals for number of aspirated oocytes and number of mature oocytes in relation to individual risk lifestyle factors in study cohorts.

		Lifestyle study cohort				UppStART study cohort			
Outcome	Variable	Crude IRR (CI)^a^	p-value	Adjusted IRR (CI)^b^	p-value	Crude IRR (CI)^a^	p-value	Adjusted IRR (CI)^b^	p-value
**Number of aspirated** **oocytes**	**BMI**	0.98 (0.97,1.00)*	0.019	0.98 (0.97, 1.00)*	0.023	0.97 (0.95, 0.99)*	0.008	0.97 (0.95, 0.99)*	0.011
	**Smoking**	0.81 (0.70, 0.94)*	0.005	0.79 (0.68, 0.93)*	0.004	0.99 (0.85, 1.16)	0.914	1.01 (0.86, 1.19)	0.865
	**Age**	1.00 (0.99, 1.01)	0.994	1.00 (0.98, 1.01)	0.893	0.98 (0.96, 0.99)*	0.010	0.98 (0.96, 1.00)*	0.017
	**Alcohol**	1.01 (0.87, 1.17)	0.873	1.04 (0.89, 1.20)	0.651	0.87 (0.73, 1.05)	0.142	0.90 (0.75, 1.08)	0.260
	**Caffeine**	1.00 (1.00, 1.00)	0.384	1.00 (1.00, 1.00)	0.885	1.00 (1.00, 1.00)	0.579	1.00 (1.00, 1.00)	0.978
	**Physical activity**	0.63 (0.31, 1.30)	0.212	0.62 (0.31, 1.26)	0.187	0.87 (0.50, 1.51)	0.617	0.75 (0.43, 1.31)	0.319
	**Depression**	1.13 (0.95, 1.34)	0.179	1.15 (0.97, 1.36)	0.099	0.88 (0.71, 1.09)	0.246	0.91 (0.73, 1.12)	0.364
**Number** **of mature oocytes**	**BMI**	0.98 (0.96, 1.00)*	0.019	0.98 (0.97, 1.00)*	0.026	0.97 (0.95, 0.99)**	0.018	0.97 (0.95, 0.99)*	0.017
	**Smoking**	0.81 (0.69, 0.94)*	0.006	0.79 (0.67, 0.93)*	0.004	1.01 (0.85, 1.18)	0.983	1.02 (0.86, 1.20)	0.743
	**Age**	1.00 (0.98, 1.01)	0.947	1.00 (0.98, 1.01)	0.753	0.98 (0.96, 0.99)*	0.010	0.98 (0.96, 1.00)*	0.022
	**Alcohol**	1.05 (0.90, 1.22)	0.529	1.08 (0.92, 1.26)	0.335	0.82 (0.69, 0.99)*	0.035	0.84 (0.70, 1.01)	0.067
	**Caffeine**	1.00 (1.00, 1.00)	0.378	1.00 (1.00, 1.00)	0.986	1.00 (1.00, 1.00)	0.889	1.00 (1.00, 1.00)	0.472
	**Physical activity**	0.71 (0.34, 1.49)	0.365	0.75 (0.34, 1.47)	0.351	0.81 (0.46, 1.44)	0.474	0.70 (0.40, 1.22)	0.207
	**Depression**	1.13 (0.94, 1.34)	0.192	1.16 (0.97, 1.38)	0.105	0.86 (0.69, 1.08)	0.196	0.89 (0.71, 1.11)	0.300

^a^ Incidence rate ratio + 95% CI for the crude univariable analysis

^b^ Incidence rate ratio + 95% CI for the adjusted analysis (adjusted for age, BMI, smoking, alcohol consumption, daily caffeine consumption, physical activity score and history of depression)

* *p*<0.05

### Association between combined lifestyle risk factors and number of aspirated oocytes

Two potential risk factors were identified in the “Lifestyle study cohort”: BMI and smoking (Table 2). In the “UppStART study” validation cohort, the variable “number of risk factors” (categorical) was found to be significant (*p* = 0.007). Women who had both risk factors had an IRR of 0.75 (95% CI: 0.61–0.94) for the number of aspirated oocytes (compared to a baseline of no risk factors), and consequently (on average) had 25% less aspirated oocytes than women without these risk factors (Table 3).

**Table 3:** Incidence rate ratio (IRR) and 95% Confidence intervals (CI) for IVF treatment outcome in association with cumulative risk lifestyles.

Outcome	Risk factors included^a^	Exposure category	Crude IRR (95% CI)^b^	p-value for comparison against reference^c^	p-value for entire categorical variable^d^	p-value for interaction with AFC^e^
**Number of aspirated** **oocytes**	BMI, Smoking	0 risk factors	Ref.		0.007	0.568
		1 risk factor	0.86 (0.73–1.00)	0.057		
		2 risk factors	0.75 (0.61–0.94)*	0.013		
**Number of mature** **oocytes**	BMI, Smoking	0 risk factors	Ref.		0.031	0.431
		1 risk factor	0.88 (0.74–1.04)	0.124		
		2 risk factors	0.78 (0.62–0.98)*	0.034		

^a^ Risk factors were identified in the “Lifestyle study” cohort

^b^ Validation models were run in the “UppSTART cohort”

^c^ Single wald p-value for the pairwise comparison of having X risk factors against the reference of 0 risk factors

^d^ Likelihood ratio test p-value for the categorical variable “number of risk factors”

^e^ Likelihood ratio test p-value for the interaction of continuous AFC with the categorical variable “number of risk factors

* p<0.05

### Association between lifestyle risk factors and number of mature oocytes

Two potential risk factors were identified in the “Lifestyle study cohort”: BMI and smoking (S1 Table). In the “UppStART study” validation cohort, the combined variable “number of risk factors” was found to have a significant association (*p* = .03), and a woman who had both risk factors had an IRR of 0.78 (95% CI: 0.62–0.98), meaning that she would (on average) have 22% fewer mature oocytes than a woman with no risk factors (Table 3).

We also ran a sensitivity analysis to see the effect of the variables 'Total FSH dosage' and 'IVF agonist protocol' on the analysis. This information was only available for the Lifestyle study cohort. We therefore recreated Table 2 for the outcomes of 'Number of aspirated oocytes' and 'Number of mature oocytes', including a third column where we included 'Total FSH dosage' and 'IVF agonist protocol' to the list of adjusted variables (S2 and S3 Tables). We found that BMI and smoking were unaffected by the inclusion of these two new confounders, highlighting the robustness of our findings.

### Interaction with AFC

While none of the cumulative risk factors had significant interactions with AFC, we did find that for the outcome of “number of mature oocytes per number of aspirated oocytes in IVF cycles” (in a model only containing BMI, AFC, and the interaction between BMI and AFC), BMI had a borderline-significant interaction with AFC in the Lifestyle study (p-value = 0.065, Table 4) and a significant interaction with AFC in the UppStART study (p-value = 0.038, Table 4). When using a model that adjusted for age, BMI, smoking, alcohol consumption, daily caffeine consumption, physical activity score and history of depression, the interaction between BMI and AFC had p-values of 0.086 and 0.045 for the Lifestyle study and UppStART study, respectively (other data not shown).

**Table 4:** Interaction between individual risk lifestyle factors with Antral Follicle Count (AFC) in affecting number of mature oocytes (IRRs with related 95% confidence intervals).

Outcome	Variable	Lifestyle study cohort					UppStART study cohort				
		Crude IRR^a^	Crude P-value^b^	Adjusted IRR^c^	Adjusted P-value^d^	Crude interaction P-value	Crude IRR^a^	Crude P-value^b^	Adjusted IRR^c^	Adjusted P-value^d^	Crude interaction P-value
Number of mature oocytes	BMI	1.00 (0.99, 1.01)	0.948	1.00 (0.99, 1.01)	0.923	**0.065**	1.00 (0.99, 1.01)	0.780	1.00 (0.99, 1.01)	0.964	**0.038**
	Smoking	0.99 (0.94, 1.04)	0.627	0.98 (0.93, 1.04)	0.583	**0.034**	1.01 (0.97, 1.06)	0.559	1.02 (0.97, 1.06)	0.498	0.743
	Age	1.00 (1.00, 1.01)	0.646	1.00 (1.00, 1.01)	0.850	0.160	1.00 (0.99, 1.00)	0.824	1.00 (1.00, 1.01)	0.883	0.766
	Alcohol	1.05 (1.00, 1.11)	0.070	1.06 (1.00, 1.12)	0.058	0.303	0.95 (0.91, 1.00)*	0.049	0.94 (0.90, 0.99)*	0.026	0.380
	Caffeine	1.00 (1.00, 1.00)	0.960	1.00 (1.00, 1.00)	0.701	0.559	1.00 (1.00, 1.00)	0.209	1.00 (1.00, 1.00)	0.157	0.664
	Physical activity	1.07 (0.83, 1.38)	0.617	1.08 (0.83, 1.40)	0.556	**0.051**	0.93 (0.81, 1.08)	0.363	0.92 (0.80, 1.07)	0.271	0.908
	Depression	0.99 (0.93, 1.05)	0.791	1.00 (0.94, 1.06)	0.954	0.850	0.99 (0.93, 1.05)	0.700	0.99 (0.93, 1.05)	0.700	0.844

^a^ Incidence rate ratio + 95% CI for the crude univariable analysis

^b^ Likelihood ratio test p-value for the crude univariable analysis

^c^ Incidence rate ratio + 95% CI for the adjusted analysis

(adjusted for age, BMI, smoking, alcohol consumption, daily caffeine consumption, physical activity score, and history of depression)

^d^ Likelihood ratio test p-value for the adjusted analysis

* p<0.05

## Discussion

Our study highlights the individual and combined impact of female lifestyle risk factors, mostly BMI and smoking, on the number of aspirated and mature oocytes in the first IVF treatment cycle. To our knowledge, this is the first study to also demonstrate a possible interaction of BMI in relation to AFC measurement when exploring its effect on the number of mature oocytes per number of aspirated oocytes in IVF cycles.

Healthy and unhealthy lifestyle behaviors tend to occur in clusters [22]. A healthy way of life can be characterized by an accumulation of multiple healthy lifestyle choices and vice versa. Therefore, researchers have lately adopted a more holistic approach to lifestyle and seek to evaluate the cumulative effect of lifestyle behaviors on health outcomes [23]. Couples who are trying to conceive are usually advised to make lifestyle modifications, but this advice may be difficult or even impossible for some women to follow [7], leading often to thoughts of self-blame as a coping mechanism [24]. It is therefore important to assess the impact of lifestyle factors and their potential interaction with ovarian reserve, with the goal to give individually tailored clinical advice. Although there are several studies addressing the impact of lifestyle risk behaviors on various IVF treatment outcomes, such as pregnancy rate or livebirth rate [3,25,26] only a few of them evaluate the association between lifestyle behaviors and number and/or quality of aspirated oocytes in the IVF setting [27].

Based on the results of the current study the effects of smoking and BMI on IVF early treatment outcomes appear to be cumulative. A higher number of negative risk lifestyle factors is correlated to a significant decrease in number of aspirated and mature oocytes. It is also interesting to note the interaction of BMI with AFC, suggesting that at appropriate levels of ovarian reserve, the impact of BMI is possibly not so important. These results could contribute to individually tailored information to couples seeking treatment.

The existing literature is inconsistent as to whether obesity affects reproduction through mechanisms involving mostly the ovaries or maybe even the uterus [27–30]. The impact regarding oocytes seems to be more pronounced; there is an increasing body of evidence demonstrating a negative correlation between number of retrieved, as well as mature (MII) oocytes in obese women implying a harmful effect of the metabolic environment on retrieved oocyte quantity and quality [27,31–34]. However, in a register- based cohort study, the effect was apparent only in first treatment-cycles, whereas no effect was observed on the following treatment-cycles (≥two) [35].

Nicotine on the other side may act as a mutagen and possible oocyte toxin. Fuentes et al., reported a proportional decrease of the number of oocytes when follicular fluid cotinine levels (i.e. a metabolite of nicotine which remains present in the body for prolonged periods) increased [36]. Furthermore, increased levels of reactive oxygen species (ROS) in follicular fluid, probably promoted by smoking exposure over a long period of time, correlate with immature oocytes and lower quality embryos, a finding that further strengthens our findings [37]. Firns et al demonstrated that basal FSH increased in tandem with years of cigarette smoking resulting, in turn, to lowered retrieved oocyte numbers and fertilization rates [10]. Our findings are in corroboration with these results.

In this study we used AMH and AFC as markers for ovarian reserve. There is still a lack of consensus regarding the established range for the low, and especially normal and above normal ovarian reserve (i.e. based solely on ovarian reserve markers without considering the total oocytes retrieved, “Bologna criteria” etc) [38]. However, there is mounting evidence to support the use of AMH (0.5 to 1.1 ng/ml) and fair evidence to support that a low antral follicle count (5–7) has moderate to high specificity as a screening test for poor ovarian response [39].

The effect of age on the number of aspirated oocytes in our study was statistically significant in the “UppStART study” validation cohort (S1 Table). This cohort includes a wider age range, (IQR; 31.0 to 37.0) and a higher age median (34.0 years) of women compared to the “Lifestyle study cohort”. In the Lifestyle cohort, which mainly included public fertility clinics, women were younger and with a narrow age range, rendering the effect of age less apparent. Despite what one would expect, median AMH was higher in the “Lifestyle study” cohort whereas median AFC was lower in that group compared to the “UppStart study” cohort (Table 1). We believe that this discrepancy could probably be due to either the fact that the “UppStart study” cohort actually had a lower BMI despite their higher age, the small sample size of the “Lifestyle study” cohort, or due to missing data regarding reported AFC in the “UppStART study” cohort. These two markers of oocyte reserve usually correlate, but they are affected by both age (favorable in the “Lifestyle study”) and somewhat by BMI (favorable in the “UppStart study”), probably differentially impacting AMH and AFC and accounting for the observed differences between the cohorts. In order to confirm the hypothesis that AMH in relation to age was similar between the two cohorts, we ran a linear regression with AMH as the outcome, a binary variable demarcating cohort ownership as the exposure, and continuous age as a covariate. We found that the cohort variable had a p-value of 0.22, which suggested that there was no evidence that AMH differed between cohorts when age was accounted for. Taking that into account, we chose to report even other variables that indirectly mirror the ovarian reserve, such as total FSH dosage used per treatment which did not differ between the two cohorts. We believe thus, that these two cohorts are clinically comparable concerning ovarian reserve. We also believe more in the clinical importance of ovarian reserve differences in different ages which have been even documented in previous research [39]. Age is of course already recognized to be the most prominent predictor for success after IVF. Female fertility peaks at the age of 22 and subsequently decreases until around the age of 38, where after it falls more rapidly until menopause [40].

Regarding alcohol consumption, a decrease in number of created embryos and number of embryos available for transfer (fresh or frozen-thawed), were detected in the “UppStART study” validation cohort (S1 Table). Surprisingly, this decrease could not be detected in the “Lifestyle study cohort”, perhaps because of socioeconomic and age differences in the two cohorts, as well as different phrasing of the specific questions in the questionnaire in the “UppStART study” validation cohort, which were more detailed with regards to amount and type of alcohol. Similar results were shown in a smaller study from Southern California which showed a decrease in the number of aspirated oocytes and number of fertilized oocytes among alcohol drinkers [41].

Furthermore, in our study, there were no statistically significant associations between caffeine or physical activity and IVF early treatment outcomes. However, caffeine consumption in this study was assessed by asking women about their coffee consumption only and not about other caffeine-containing beverages. Caffeine consumption has been shown to be negatively associated with the number of oocytes collected [42] but Abadia et al found no further influence of caffeine on intermediate or clinical endpoints of ART [43]. In addition, we did not ask about specific types of physical activity, but rather ascertained the number of hours of activity per week for all types of vigorous or moderate physical activity combined. More specifically, increasing amounts of exercise appear to have a variable effect on IVF outcomes [44], but certain categories of exercise may adversely affect IVF-related pregnancy rates [26,45].

### Limitations and strengths

Limitations include the self-reported data collected through study participant questionnaires for some of the lifestyle variables. It is well known for example that participants tend to underreport non-socially desirable behaviors, such as alcohol intake and smoking, possibly resulting in misclassification of some individuals. However, that is not the case for the details of IVF treatment, where all data were retrieved from participants’ medical records retrospectively and are thus highly reliable. This study did not include the corresponding male variables on lifestyle, which is a limitation since fertilization rates are affected by semen quality, which is in turn strongly associated to lifestyle behaviors in the partner. Furthermore, the relatively small sample size, especially in the “Lifestyle study cohort”, might introduce some problems with statistical power. This might be reflected in the borderline statistical significance of some of the results, where, on the other hand, notable and stable trends are demonstrated. Despite these shortcomings, this study is one of the largest on the field evaluating lifestyle factors, their interaction and possible impact on assisted reproductive early treatment outcomes, and uses a larger, validation cohort from the same geographic area to confirm the study findings. Participation rate was higher than in many other studies in the field [46].

Among the strengths of the study is the prospective design with complete follow up and the simultaneous inclusion of several lifestyle risk variables. Furthermore, by only exploring the first fresh IVF treatment cycle of every patient who met the inclusion criteria, the negative effects resulting from the history of repeatedly failed or cryopreserved cycles were minimized. In addition, we used one cohort for hypothesis generation, and another to validate our findings, adding strength to the validity of our findings. The sample of women with fertility problems was drawn from both public and private fertility clinics, which makes the sample representative regarding women undergoing fertility treatment in mid-Sweden and minimizes the risk for selection bias.

## Conclusions

The findings of this study point both to an individual as well as a cumulative effect of smoking and BMI on the number of aspirated oocytes and the number of mature oocytes in fresh IVF treatment-cycles. AFC might modify associations between BMI and the proportion of mature oocytes in relation to the total number of aspirated oocytes. These negative lifestyle factors are easy to detect at an early stage of the assessment process and might allow for optimization of the early treatment outcome. Whether women may be able to improve the outcome of fertility treatment by quitting smoking and losing weight must be further investigated.

## Supporting information

S1 Table Incidence rate ratio (IRR) and 95% Confidence intervals (CI) for IVF treatment outcomes in relation to risk lifestyle factors with and without denominator. (DOCX)

S2 Table Sensitivity analysis for the outcome number of aspirated oocytes including even FSH total dose and IVF protocol in the model. (DOCX)

S3 Table Sensitivity analysis for the outcome number of mature oocytes including FSH total dose and IVF agonist protocol in the model. (DOCX)

S1 Questionnaire Lifestyle study- Swedish. (PDF)

S2 Questionnaire Lifestyle study- English. (PDF)

## References

[1] Thoma ME, McLain AC, Louis JF, King RB, Trumble AC, Sundaram R, et al Prevalence of infertility in the United States as estimated by the current duration approach and a traditional constructed approach. Fertil Steril. Elsevier Inc.; 2013;99: 1324–1331.e1. 10.1016/j.fertnstert.2012.11.037 23290741 PMC3615032

[2] McLernon DJ, Maheshwari A, Lee AJ, Bhattacharya S, Nelson SM, Lawlor DA. Cumulative live birth rates after one or more complete cycles of IVF: A population-based study of linked cycle data from 178 898 women. Hum Reprod. 2016;31: 572–581. 10.1093/humrep/dev336 26783243

[3] Homan GF, Davies M, Norman R. The impact of lifestyle factors on reproductive performance in the general population and those undergoing infertility treatment: A review. Hum Reprod Update. 2007;13: 209–223. 10.1093/humupd/dml056 17208948

[4] Stephenson J, Heslehurst N, Hall J, Schoenaker DAJM, Hutchinson J, Cade JE, et al Series Preconception health 1 Before the beginning: nutrition and lifestyle in the preconception period and its importance for future health. Lancet. Elsevier Ltd; 2018;6736: 1–12. 10.1016/S0140-6736(18)30311-8 PMC6075697 29673873

[5] Anderson K, Norman R, Middleton P. Preconception lifestyle advice for people with subfertility—Cochrane Database of Systematic Reviews—Anderson—Wiley Online Library. Cochrane Libr. 2010;4: 1–33. 10.1002/14651858.CD008189.pub2 20393968

[6] Anderson K, Nisenblat V, Norman R. Lifestyle factors in people seeking infertility treatment—A review: Invited Review. Aust New Zeal J Obstet Gynaecol. 2010;50: 8–20. 10.1111/j.1479-828X.2009.01119.x 20218991

[7] ESHRE Task Force on Ethics and Law., Dondorp W, De Wert G, Pennings G, Shenfield F, Devroey P, et al Lifestyle-related factors and access to medically assisted reproduction. Hum Reprod. England; 2010;25: 578–583. 10.1093/humrep/dep458 20085914

[8] Rossi B, Abusief M, Missmer S. Modifiable Risk Factors and Infertility What Are the Connections? Am J Lifestyle. 2014;10: 220–231. 10.1177/1559827614558020.Modifiable PMC5007064 27594813

[9] Lintsen AMEE Pasker-de Jong PCMM, de Boer EJ Burger CW, Jansen CAMM Braat DDMM, et al Effects of subfertility cause, smoking and body weight on the success rate of IVF. Hum Reprod. 2005;20: 1867–1875. 10.1093/humrep/deh898 15817580

[10] Firns S, Cruzat VF, Keane KN, Joesbury KA, Lee AH, Newsholme P, et al The effect of cigarette smoking, alcohol consumption and fruit and vegetable consumption on IVF outcomes: a review and presentation of original data. Reprod Biol Endocrinol. Reproductive Biology and Endocrinology; 2015;13: 134 10.1186/s12958-015-0133-x 26669322 PMC4681150

[11] Sunkara SK, Rittenberg V, Raine-Fenning N, Bhattacharya S, Zamora J, Coomarasamy A. Association between the number of eggs and live birth in IVF treatment: An analysis of 400 135 treatment cycles. Hum Reprod. 2011;26: 1768–1774. 10.1093/humrep/der106 21558332

[12] Hariton E, Kim K, Mumford SL, Palmor M, Bortoletto P, Cardozo ER, et al Total number of oocytes and zygotes are predictive of live birth pregnancy in fresh donor oocyte in vitro fertilization cycles. Fertil Steril. Elsevier Inc.; 2017;108: 262–268. 10.1016/j.fertnstert.2017.05.021 28601410 PMC5545054

[13] Joelsson LS, Berglund A, Wånggren K, Lood M, Rosenblad A, Tydén T. Do subfertile women adjust their habits when trying to conceive? Ups J Med Sci. 2016;9734: 1–8. 10.1080/03009734.2016.1176094 27216564 PMC4967265

[14] Alpha Scientists in Reproductive Medicine, Embryology ESIG of. The Istanbul consensus workshop on embryo assessment: proceedings. Hum Reprod. 2011;0: 1–14. 10.1093/humrep/der037

[15] Study UppstART, Uppsala-Stockholm Assisted Reproductive Techniques. Med Vetensk. 2011; Available: https://ki.se/meb/uppstart

[16] Carolyn E Cesta, Anna L Johansson, Julius Hreinsson, Kenny A Rodriguez Wallberg, Jan I Olofsson, Jan Holte, kan Wramsby, Margareta Wramsby, Sven Cnattingius AS& AN-I, Cesta CE, Johansson AL V, Hreinsson J, Rodriguez‐Wallberg KA, Olofsson JI, et al A prospective investigation of perceived stress, infertility‐related stress, and cortisol levels in women undergoing in vitro fertilization: influence on embryo quality and clinical pregnancy rate. Acta Obstet Gynecol Scand. Wiley Online Library; 2018; 10.1111/aogs.13280 29250769

[17] Bexelius C, Löf M, Sandin S, Lagerros YT, Forsum E, Litton JE. Measures of physical activity using cell phones: Validation using criterion methods. J Med Internet Res. 2010;12 10.2196/jmir.1298 20118036 PMC2821583

[18] Schenker N TJ. Partially parametric techniques for multiple imputation. Comput Stat Data Anal. 1996;22: 425–446. 10.1016/0167-9473(95)00057-7

[19] Heitjan DF, Little RJA. Multiple Imputation for the Fatal Accident Reporting System. J R Stat Soc Ser C (Applied Stat. [Wiley, Royal Statistical Society]; 1991;40: 13–29. 10.2307/2347902

[20] Buuren S van, Groothuis-Oudshoorn K. mice: Multivariate Imputation by Chained Equations in R. J Stat Softw. 2011;45: 1–67. 10.18637/jss.v045.i03

[21] RUBIN DB. Multiple Imputation for Nonresponse in Surveys. New York JOHN WILEY SONS 1987; 10.1002/9780470316696

[22] Meader N, King K, Moe-byrne T, Wright K, Graham H, Petticrew M, et al A systematic review on the clustering and co-occurrence of multiple risk behaviours. BMC Public Health. BMC Public Health; 2016; 1–9. 10.1186/s12889-015-2639-8 27473458 PMC4966774

[23] Buck D, Frosini F. Clustering of unhealthy behaviours over time—Implications for policy and practice. Kings Fund. 2012; 1–24. Available: www.kingsfund.org.uk

[24] Rossi B V., Bressler LH, Correia KF, Lipskind S, Hornstein MD, Missmer SA. Lifestyle and in vitro fertilization: what do patients believe? Fertil Res Pract. Fertility Research and Practice; 2016;2: 11 10.1186/s40738-016-0026-5 28620538 PMC5424337

[25] Rooney KL, Domar AD. The impact of lifestyle behaviors on infertility treatment outcome. Curr Opin Obstet Gynecol. 2014;26: 181–5. 10.1097/GCO.0000000000000069 24752004

[26] Hornstein MD. Lifestyle and IVF Outcomes. Reprod Sci. 2016;23: 1626–1629. 10.1177/1933719116667226 27609400

[27] Zhang JJ, Feret M, Chang L, Yang M, Merhi Z. Obesity adversely impacts the number and maturity of oocytes in conventional IVF not in minimal stimulation IVF. Gynecol Endocrinol. 2015;31: 409–13. 10.3109/09513590.2015.1014785 25856299

[28] Luke B, Brown MB, Stern JE, Missmer SA, Fujimoto VY, Leach R. Female obesity adversely affects assisted reproductive technology (ART) pregnancy and live birth rates. Hum Reprod. England; 2011;26: 245–252. 10.1093/humrep/deq306 21071489

[29] Bellver J, Pellicer A, García-Velasco JA, Ballesteros A, Remohí J, Meseguer M. Obesity reduces uterine receptivity: Clinical experience from 9,587 first cycles of ovum donation with normal weight donors. Fertil Steril. 2013;100 10.1016/j.fertnstert.2013.02.056 23830106

[30] Cardozo E, Pavone ME, Hirshfeld-Cytron JE. Metabolic syndrome and oocyte quality. Trends Endocrinol Metab. United States; 2011;22: 103–109. 10.1016/j.tem.2010.12.002 21277789

[31] Cardozo ER, Karmon AE, Gold J, Petrozza JC, Styer AK. Reproductive outcomes in oocyte donation cycles are associated with donor BMI. Hum Reprod. 2016;31: 385–392. 10.1093/humrep/dev298 26677960

[32] Wittemer C, Ohl J, Bailly M, Bettahar-Lebugle K, Nisand I. Does body mass index of infertile women have an impact on IVF procedure and outcome? J Assist Reprod Genet. 2000;17: 547–52. 10.1023/A:1026477628723 11209534 PMC3455453

[33] Esinler I, Bozdag G, Yarali H. Impact of isolated obesity on ICSI outcome. Reprod Biomed Online. Reproductive Healthcare Ltd, Duck End Farm, Dry Drayton, Cambridge CB23 8DB, UK; 2008;17: 583–587. 10.1016/S1472-6483(10)60249-0 18854116

[34] Zhang D, Zhu Y, Gao H, Zhou B, Zhang R, Wang T, et al Overweight and obesity negatively affect the outcomes of ovarian stimulation and in vitro fertilisation: a cohort study of 2628 Chinese women. Gynecol Endocrinol. 2010;26: 325–32. 10.3109/09513591003632100 20192898

[35] Christensen MW, Ingerslev HJ, Degn B, Kesmodel US. Effect of female body mass index on oocyte quantity in fertility treatments (IVF): Treatment cycle number is a possible effect modifier. A register-based cohort study. PLoS One. 2016;11: 1–15. 10.1371/journal.pone.0163393 27654907 PMC5031400

[36] Fuentes A, Munoz A, Barnhart K, Arguello B, Diaz M, Pommer R. Recent cigarette smoking and assisted reproductive technologies outcome. Fertil Steril. United States; 2010;93: 89–95. 10.1016/j.fertnstert.2008.09.073 18973890

[37] Singh AK, Chattopadhyay R, Chakravarty B, Chaudhury K. Markers of oxidative stress in follicular fluid of women with endometriosis and tubal infertility undergoing IVF. Reprod Toxicol. Elsevier Inc.; 2013;42: 116–124. 10.1016/j.reprotox.2013.08.005 23994512

[38] Ferraretti AP, La Marca A, Fauser BCJM, Tarlatzis B, Nargund G, Gianaroli L. ESHRE consensus on the definition of ‘poor response to ovarian stimulation for in vitro fertilization: The Bologna criteria. Hum Reprod. 2011;26: 1616–1624. 10.1093/humrep/der092 21505041

[39] Pfeifer S, Butts S, Dumesic D, Fossum G, Giudice L, Gracia C, et al Testing and interpreting measures of ovarian reserve: A committee opinion. Fertil Steril. American Society for Reproductive Medicine; 2015;103: e9–e17. 10.1016/j.fertnstert.2014.12.093 25585505

[40] Dunson DB, Baird DD, Colombo B. Increased infertility with age in men and women. Obs Gynecol. 2004;103. 10.1097/01.AOG.0000100153.24061.45 14704244

[41] Klonoff-Cohen H, Lam-Kruglick P, Gonzalez C. Effects of maternal and paternal alcohol consumption on the success rates of in vitro fertilization and gamete intrafallopian transfer. Fertil Steril. 2003;79: 330–339. 10.1016/s0015-0282(02)04582-x 12568842

[42] Al-Saleh I, El-Doush I, Grisellhi B, Coskun S. The effect of caffeine consumption on the success rate of pregnancy as well various performance parameters of in-vitro fertilization treatment. Med Sci Monit. United States; 2010;16: CR598-R605. 881297 [pii] 21119578

[43] Abadia L, Chiu Y-H, Williams PL, Toth TL, Souter I, Hauser R, et al The association between pre-treatment maternal alcohol and caffeine intake and outcomes of assisted reproduction in a prospectively followed cohort. Hum Reprod. England; 2017;32: 1846–1854. 10.1093/humrep/dex237 28854726 PMC5850490

[44] Evenson KR, Calhoun KC, Herring AH, Pritchard D, Wen F, Steiner AZ. Association of physical activity in the past year and immediately after in vitro fertilization on pregnancy. Fertil Steril. Elsevier Inc.; 2014;101: 1047–1054.e5. 10.1016/j.fertnstert.2013.12.041 24524834 PMC3982290

[45] Morris SN, Missmer SA, Cramer DW, Powers RD, McShane PM, Hornstein MD. Effects of lifetime exercise on the outcome of in vitro fertilization. Obs Gynecol. 2006;108. 10.1097/01.AOG.0000235704.45652.0b 17012457

[46] Wenemark M. The respondent ‘ s perspective in health-related surveys The role of motivation. 2010. http://liu.diva-portal.org/smash/get/diva2:355603/FULLTEXT01.pdf

