# Supplementary material for: Investigating the effect of lifestyle risk factors upon number of aspirated and mature oocytes in in vitro fertilization cycles: Interaction with antral follicle count
Source: PLoS One. 2019 Aug 16;14(8):e0221015. doi: 10.1371/journal.pone.0221015 (PMC6697332; doi:10.1371/journal.pone.0221015)
Supplement: S1 Table — (DOCX) [file pone.0221015.s001.docx]

**S1 Table.**

Incidence rate ratio (IRR) and 95% Confidence intervals (CI) for IVF treatment outcomes in relation to risk lifestyle factors with and without denominator.

|  |  | **Lifestyle study cohort** | | | | | **UppStART study cohort** | | | | |
| --- | --- | --- | --- | --- | --- | --- | --- | --- | --- | --- | --- |
|  |  |  | | | | |  | | | | |
| **Outcome** | **Variable** | **Crude IRR^a^** | **Crude**  **P-value^b^** | **Adjusted IRR^c^** | **Adjusted**  **P-value^d^** | **Crude interaction**  **P-value** | **Crude_IRR^a^** | **Crude**  **p-value^b^** | **Adjusted IRR^c^** | **Adjusted**  **p-value^d^** | **Crude interaction**  **p-value** |
| Number  of aspirated oocytes | BMI | 0.98 (0.97, 1.00)*^e^ | 0.019 | 0.98 (0.97, 1.00)* | 0.023 | 0.731 | 0.97 (0.95, 0.99)* | 0.008 | 0.97 (0.95, 0.99)* | 0.011 | 0.603 |
|  | smoking | 0.81 (0.70, 0.94)* | 0.005 | 0.79 (0.68, 0.93)* | 0.004 | 0.751 | 0.99 (0.85, 1.16) | 0.914 | 1.01 (0.86, 1.19) | 0.865 | 0.375 |
|  | age | 1.00 (0.99, 1.01) | 0.994 | 1.00 (0.98, 1.01) | 0.889 | 0.027 | 0.98 (0.96, 0.99)* | 0.010 | 0.98 (0.96, 1.00)* | 0.017 | 0.392 |
|  | alcohol | 1.01 (0.87, 1.17) | 0.873 | 1.04 (0.89, 1.20) | 0.651 | 0.038 | 0.87 (0.73, 1.05) | 0.142 | 0.90 (0.75, 1.08) | 0.260 | 0.928 |
|  | caffeine | 1.00 (1.00, 1.00) | 0.384 | 1.00 (1.00, 1.00) | 0.885 | 0.170 | 1.00 (1.00, 1.00) | 0.579 | 1.00 (1.00, 1.00) | 0.978 | 0.678 |
|  | physical activity | 0.63 (0.31, 1.30) | 0.212 | 0.62 (0.31, 1.26) | 0.187 | 0.793 | 0.87 (0.50, 1.51) | 0.617 | 0.75 (0.43, 1.31) | 0.880 | 0.796 |
|  | depression | 1.13 (0.95, 1.34) | 0.179 | 1.15 (0.97, 1.36) | 0.099 | 0.165 | 0.88 (0.71, 1.09) | 0.246 | 0.91 (0.73, 1.12) | 0.364 | 0.497 |
|  |  |  |  |  |  |  |  |  |  |  |  |
| Number  of mature oocytes | BMI | 0.98 (0.96, 1.00)* | 0.019 | 0.98 (0.97, 1.00)* | 0.026 | 0.361 | 0.97 (0.95, 0.99)* | 0.018 | 0.97 (0.95, 0.99)* | 0.017 | 0.304 |
|  | smoking | 0.81 (0.69, 0.94)* | 0.006 | 0.79 (0.67, 0.93)* | 0.004 | 0.228 | 1.01 (0.85, 1.18) | 0.983 | 1.02 (0.86, 1.20) | 0.743 | 0.667 |
|  | age | 1.00 (0.98, 1.01) | 0.947 | 1.00 (0.98, 1.01) | 0.753 | 0.006 | 0.98 (0.96, 0.99)* | 0.010 | 0.98 (0.96, 1.00)* | 0.022 | 0.431 |
|  | alcohol | 1.05 (0.90, 1.22) | 0.529 | 1.08 (0.92, 1.26) | 0.335 | 0.114 | 0.82 (0.69, 0.99)* | 0.035 | 0.84 (0.70, 1.01) | 0.067 | 0.850 |
|  | caffeine | 1.00 (1.00, 1.00) | 0.378 | 1.00 (1.00, 1.00) | 0.986 | 0.120 | 1.00 (1.00, 1.00) | 0.889 | 1.00 (1.00, 1.00) | 0.472 | 0.593 |
|  | physical activity | 0.71 (0.34, 1.49) | 0.365 | 0.75 (0.34, 1.47) | 0.351 | 0.690 | 0.81 (0.46, 1.44) | 0.474 | 0.70 (0.40, 1.22) | 0.207 | 0.946 |
|  | depression | 1.13 (0.94, 1.34) | 0.192 | 1.16 (0.97, 1.38) | 0.105 | 0.255 | 0.86 (0.69, 1.08) | 0.196 | 0.89 (0.71, 1.11) | 0.300 | 0.806 |
|  |  |  |  |  |  |  |  |  |  |  |  |
| Number  of created embryos | BMI | 0.97 (0.95, 0.99)* | 0.006 | 0.97 (0.95, 0.99)* | 0.009 | 0.229 | 0.97 (0.94, 1.00)* | 0.030 | 0.97 (0.94, 1.00)* | 0.024 | 0.612 |
|  | smoking | 0.84 (0.69, 1.02) | 0.077 | 0.81 (0.66, 0.99)* | 0.040 | 0.321 | 1.04 (0.86, 1.27) | 0.663 | 1.06 (0.87, 1.29) | 0.554 | 0.493 |
|  | age | 1.00 (0.98, 1.02) | 0.787 | 0.99 (0.97, 1.01) | 0.498 | 0.024 | 0.99 (0.97, 1.01) | 0.399 | 0.99 (0.97, 1.01) | 0.536 | 0.147 |
|  | alcohol | 1.19 (0.98, 1.45) | 0.088 | 1.22 (1.00, 1.50) | 0.054 | 0.031 | 0.80 (0.65, 0.99)* | 0.042 | 0.79 (0.64, 0.98)* | 0.036 | 0.539 |
|  | caffeine | 1.00 (1.00, 1.00) | 0.564 | 1.00 (1.00, 1.00) | 0.987 | 0.486 | 1.00 (1.00, 1.00) | 0.861 | 1.00 (1.00, 1.00) | 0.467 | 0.723 |
|  | physical activity | 0.54 (0.20, 1.40) | 0.205 | 0.57 (0.22, 1.47) | 0.247 | 0.769 | 0.64 (0.33, 1.26) | 0.198 | 0.58 (0.30, 1.14) | 0.117 | 0.939 |
|  | depression | 1.06 (0.84, 1.33) | 0.652 | 1.09 (0.87, 1.36) | 0.461 | 0.211 | 0.91 (0.70, 1.18) | 0.484 | 0.93 (0.72, 1.21) | 0.589 | 0.431 |
|  |  |  |  |  |  |  |  |  |  |  |  |
| Number  of utilizable embryos | BMI | 0.96 (0.94, 0.99)* | 0.003 | 0.96 (0.94, 0.99)* | 0.004 | 0.160 | 0.98 (0.95, 1.01) | 0.163 | 0.98 (0.95, 1.01) | 0.189 | 0.658 |
|  | smoking | 0.93 (0.74, 1.16) | 0.504 | 0.89 (0.71, 1.11) | 0.299 | 0.622 | 1.06 (0.85, 1.32) | 0.610 | 1.14 (0.90, 1.43) | 0.275 | 0.561 |
|  | age | 0.99 (0.97, 1.01) | 0.369 | 0.99 (0.96, 1.01) | 0.236 | 0.011 | 0.98 (0.96, 1.01) | 0.120 | 0.98 (0.96, 1.01) | 0.225 | 0.314 |
|  | alcohol | 1.12 (0.89, 1.41) | 0.336 | 1.13 (0.89, 1.43) | 0.312 | 0.006 | 0.73 (0.58, 0.92)* | 0.009 | 0.73 (0.57, 0.93)* | 0.012 | 0.496 |
|  | caffeine | 1.00 (1.00, 1.00) | 0.968 | 1.00 (1.00, 1.00) | 0.516 | 0.323 | 1.00 (1.00, 1.00) | 0.591 | 1.00 (1.00, 1.00) | 0.871 | 0.684 |
|  | physical activity | 0.46 (0.15, 1.39) | 0.171 | 0.52 (0.18, 1.51) | 0.230 | 0.839 | 1.15 (0.52, 2.53) | 0.728 | 1.00 (0.46, 2.20) | 0.999 | 0.973 |
|  | depression | 1.07 (0.82, 1.40) | 0.632 | 1.09 (0.84, 1.41) | 0.519 | 0.880 | 0.84 (0.62, 1.15) | 0.275 | 0.87 (0.64, 1.19) | 0.377 | 0.697 |
|  |  |  |  |  |  |  |  |  |  |  |  |
|  |  |  |  |  |  |  |  |  |  |  |  |
| Number  of mature oocytes – denominator aspirated oocyte | BMI | 1.00 (0.99, 1.01) | 0.948 | 1.00 (0.99, 1.01) | 0.923 | 0.065 | 1.00 (0.99, 1.01) | 0.780 | 1.00 (0.99, 1.01) | 0.964 | 0.038 |
|  | Smoking | 0.99 (0.94, 1.04) | 0.627 | 0.98 (0.93, 1.04) | 0.583 | 0.034 | 1.01 (0.97, 1.06) | 0.559 | 1.02 (0.97, 1.06) | 0.498 | 0.743 |
|  | Age | 1.00 (1.00, 1.01) | 0.646 | 1.00 (1.00, 1.01) | 0.850 | 0.160 | 1.00 (0.99, 1.00) | 0.824 | 1.00 (1.00, 1.01) | 0.883 | 0.766 |
|  | alcohol | 1.05 (1.00, 1.11) | 0.070 | 1.06 (1.00, 1.12) | 0.058 | 0.303 | 0.95 (0.91, 1.00)* | 0.049 | 0.94 (0.90, 0.99)* | 0.026 | 0.380 |
|  | caffeine | 1.00 (1.00, 1.00) | 0.960 | 1.00 (1.00, 1.00) | 0.701 | 0.559 | 1.00 (1.00, 1.00) | 0.209 | 1.00 (1.00, 1.00) | 0.157 | 0.664 |
|  | Physical activity | 1.07 (0.83, 1.38) | 0.617 | 1.08 (0.83, 1.40) | 0.556 | 0.051 | 0.93 (0.81, 1.08) | 0.363 | 0.92 (0.80, 1.07) | 0.271 | 0.908 |
|  | depression_ever | 0.99 (0.93, 1.05) | 0.791 | 1.00 (0.94, 1.06) | 0.954 | 0.850 | 0.99 (0.93, 1.05) | 0.700 | 0.99 (0.93, 1.05) | 0.700 | 0.844 |
|  |  |  |  |  |  |  |  |  |  |  |  |
| Number  Of created embryos  -denominator aspirated oocyte  Number  Of used embryos- denominator aspirated oocyte | BMI | 0.99 (0.97, 1.00) | 0.182 | 0.99 (0.97, 1.01) | 0.195 | 0.054 | 1.00 (0.98, 1.01) | 0.855 | 1.00 (0.98, 1.01) | 0.659 | 0.884 |
|  | Smoking | 1.04 (0.90, 1.20) | 0.633 | 1.02 (0.88, 1.18) | 0.799 | 0.312 | 1.05 (0.94, 1.17) | 0.398 | 1.04 (0.92, 1.17) | 0.558 | 0.637 |
|  | Age | 1.00 (0.98, 1.01) | 0.712 | 1.00 (0.98, 1.01) | 0.575 | 0.289 | 1.01 (1.00, 1.03)* | 0.025 | 1.02 (1.00, 1.03)* | 0.018 | 0.264 |
|  | alcohol | 1.18 (1.02, 1.36)* | 0.029 | 1.18 (1.01, 1.38)* | 0.035 | 0.290 | 0.93 (0.82, 1.05) | 0.249 | 0.88 (0.77, 1.00) | 0.054 | 0.671 |
|  | caffeine | 1.00 (1.00, 1.00) | 0.932 | 1.00 (1.00, 1.00) | 0.802 | 0.728 | 1.00 (1.00, 1.00) | 0.533 | 1.00 (1.00, 1.00) | 0.372 | 0.964 |
|  | Physical activity | 0.84 (0.43, 1.65) | 0.607 | 0.93 (0.47, 1.84) | 0.827 | 0.904 | 0.74 (0.50, 1.10) | 0.139 | 0.76 (0.52, 1.13) | 0.175 | 0.722 |
|  | depression | 0.94 (0.80, 1.11) | 0.475 | 0.94 (0.80, 1.12) | 0.512 | 0.589 | 1.04 (0.90, 1.21) | 0.573 | 1.03 (0.88, 1.20) | 0.730 | 0.412 |
|  |  |  |  |  |  |  |  |  |  |  |  |
|  | BMI | 0.98 (0.96, 1.00) | 0.090 | 0.98 (0.96, 1.00) | 0.082 | 0.071 | 1.01 (0.99, 1.04) | 0.272 | 1.01 (0.99, 1.04) | 0.234 | 0.885 |
|  | Smoking | 1.14 (0.94, 1.38) | 0.180 | 1.10 (0.90, 1.35) | 0.346 | 0.711 | 1.09 (0.92, 1.29) | 0.332 | 1.12 (0.94, 1.33) | 0.202 | 0.870 |
|  | Age | 0.99 (0.97, 1.01) | 0.307 | 0.99 (0.97, 1.01) | 0.257 | 0.146 | 1.01 (0.99, 1.03) | 0.396 | 1.01 (0.99, 1.03) | 0.208 | 0.639 |
|  | alcohol | 1.11 (0.91, 1.35) | 0.291 | 1.08 (0.88, 1.33) | 0.442 | 0.080 | 0.84 (0.71, 1.01) | 0.067 | 0.82 (0.68, 0.99)* | 0.039 | 0.750 |
|  | caffeine | 1.00 (1.00, 1.00) | 0.440 | 1.00 (1.00, 1.00) | 0.460 | 0.896 | 1.00 (1.00, 1.00) | 0.771 | 1.00 (1.00, 1.00) | 0.687 | 0.220 |
|  | Physical activity | 0.69 (0.28, 1.73) | 0.435 | 0.80 (0.32, 2.01) | 0.628 | 0.983 | 1.30 (0.72, 2.32) | 0.383 | 1.31 (0.73, 2.35) | 0.359 | 0.868 |
|  | depression | 0.95 (0.75, 1.19) | 0.638 | 0.93 (0.74, 1.17) | 0.547 | 0.445 | 1.02 (0.81, 1.30) | 0.843 | 1.00 (0.79, 1.28) | 0.968 | 0.875 |
|  |  |  |  |  |  |  |  |  |  |  |  |

^a^ Incidence rate ratio + 95% CI for the crude univariable analysis

^b^ Likelihood ratio test p-value for the crude univariable analysis

^c^ Incidence rate ratio + 95% CI for the adjusted analysis

(adjusted for age, BMI, smoking, alcohol consumption, daily caffeine consumption, physical activity score, and history of depression)

^d^ Likelihood ratio test p-value for the adjusted analysis

^e^ p<0.05
