# Supplementary material for: Investigating the effect of lifestyle risk factors upon number of aspirated and mature oocytes in in vitro fertilization cycles: Interaction with antral follicle count
Source: PLoS One. 2019 Aug 16;14(8):e0221015. doi: 10.1371/journal.pone.0221015 (PMC6697332; doi:10.1371/journal.pone.0221015)
Supplement: S2 Table — (DOCX) [file pone.0221015.s002.docx]

S2 Table. Sensitivity analysis for the outcome number of aspirated oocytes including Total FSH dose and IVF agonist protocol in the model.

|  | Lifestyle study | | | | | |
| --- | --- | --- | --- | --- | --- | --- |
| Lifestyle factor | Crude IRR | Crude P-value | Adjusted IRR | Adjusted P-value | Adjusted IRR (including variables FSH + IVF protocol) | Adjusted (including variables FSH + IVF protocol)  P-value |
| BMI | 0.98 (0.97, 1.00)* | 0.019 | 0.98 (0.97, 1.00)* | 0.023 | 0.99 (0.98, 1.00)* | 0.010 |
| Smoking | 0.81 (0.70, 0.94)* | 0.005 | 0.79 (0.68, 0.93)* | 0.004 | 0.78 (0.71, 0.86)* | 0.000 |
| Age | 1.00 (0.99, 1.01) | 0.994 | 1.00 (0.98, 1.01) | 0.893 | 1.01 (1.00, 1.02) | 0.063 |
| Alcohol | 1.01 (0.87, 1.17) | 0.873 | 1.04 (0.89, 1.20) | 0.651 | 1.04 (0.95, 1.15) | 0.365 |
| Caffeine | 1.00 (1.00, 1.00) | 0.384 | 1.00 (1.00, 1.00) | 0.885 | 1.00 (1.00, 1.00) | 0.639 |
| Physical activity | 0.63 (0.31, 1.30) | 0.212 | 0.62 (0.31, 1.26) | 0.187 | 0.58 (0.37, 0.91)* | 0.020 |
| Depression | 1.13 (0.95, 1.34) | 0.179 | 1.15 (0.97, 1.36) | 0.099 | 1.15 (1.04, 1.28)* | 0.008 |
| Total FSH dose/1000 |  |  |  |  | 0.84 (0.79, 0.89)* | 0.000 |
| Agonist protocol |  |  |  |  | 1.00 (0.91, 1.11) | 0.924 |
|  |  |  |  |  |  |  |

^*^ p<0.05
