# Supplementary material for: Investigating the effect of lifestyle risk factors upon number of aspirated and mature oocytes in in vitro fertilization cycles: Interaction with antral follicle count
Source: PLoS One. 2019 Aug 16;14(8):e0221015. doi: 10.1371/journal.pone.0221015 (PMC6697332; doi:10.1371/journal.pone.0221015)
Supplement: S3 Table — (DOCX) [file pone.0221015.s003.docx]

S3 Table. Sensitivity analysis for the outcome number of mature oocytes including Total FSH dose and IVF agonist protocol in the model.

| Lifestyle factor |  |  | Lifestyle study |  |  |  |
| --- | --- | --- | --- | --- | --- | --- |
|  | Crude IRR | Crude  P-value | Adjusted IRR | Adjusted  P-value | Adjusted IRR (including variables FSH + IVF protocol) | Adjusted (including variables FSH + IVF protocol)  P-value |
| BMI | 0.98 (0.96, 1.00)* | 0.019 | 0.98 (0.97, 1.00)* | 0.026 | 0.99 (0.98, 1.00)* | 0.021 |
| Smoking | 0.81 (0.69, 0.94)* | 0.006 | 0.79 (0.67, 0.93)* | 0.004 | 0.77 (0.70, 0.86)* | 0.000 |
| Age | 1.00 (0.98, 1.01) | 0.947 | 1.00 (0.98, 1.01) | 0.753 | 1.01 (1.00, 1.02) | 0.096 |
| Alcohol | 1.05 (0.90, 1.22) | 0.529 | 1.08 (0.92, 1.26) | 0.335 | 1.09 (0.99, 1.21) | 0.090 |
| Caffeine | 1.00 (1.00, 1.00) | 0.378 | 1.00 (1.00, 1.00) | 0.986 | 1.00 (1.00, 1.00) | 0.757 |
| Physical activity | 0.71 (0.34, 1.49) | 0.365 | 0.70 (0.34, 1.47) | 0.351 | 0.65 (0.40, 1.05) | 0.081 |
| Depression | 1.13 (0.94, 1.34) | 0.192 | 1.16 (0.97, 1.38) | 0.105 | 1.15 (1.03, 1.29)* | 0.016 |
| Total FSH dose/1000 |  | |  |  | 0.83 (0.78, 0.89)* | 0.000 |
| Agonist protocol |  |  |  |  | 0.93 (0.84, 1.03) | 0.187 |
|  |  |  |  |  |  |  |

^*^ p<0.05
