# Supplementary material for: Investigating the effect of lifestyle risk factors upon number of aspirated and mature oocytes in in vitro fertilization cycles: Interaction with antral follicle count
Source: PLoS One. 2019 Aug 16;14(8):e0221015. doi: 10.1371/journal.pone.0221015 (PMC6697332; doi:10.1371/journal.pone.0221015)
Supplement: S1 Questionnaire — (PDF) [file pone.0221015.s004.pdf]

LIVSSTIL OCH INFERTILITET

EN STUDIE OM FRUKTSAMHET

INSTITUTIONEN FÖR FOLKHÄLSO- OCH VÅRDVETENSKAP

INSTITUTIONEN FÖR KVINNORS OCH BARNS HÄLSA

UPPSALA UNIVERSITET

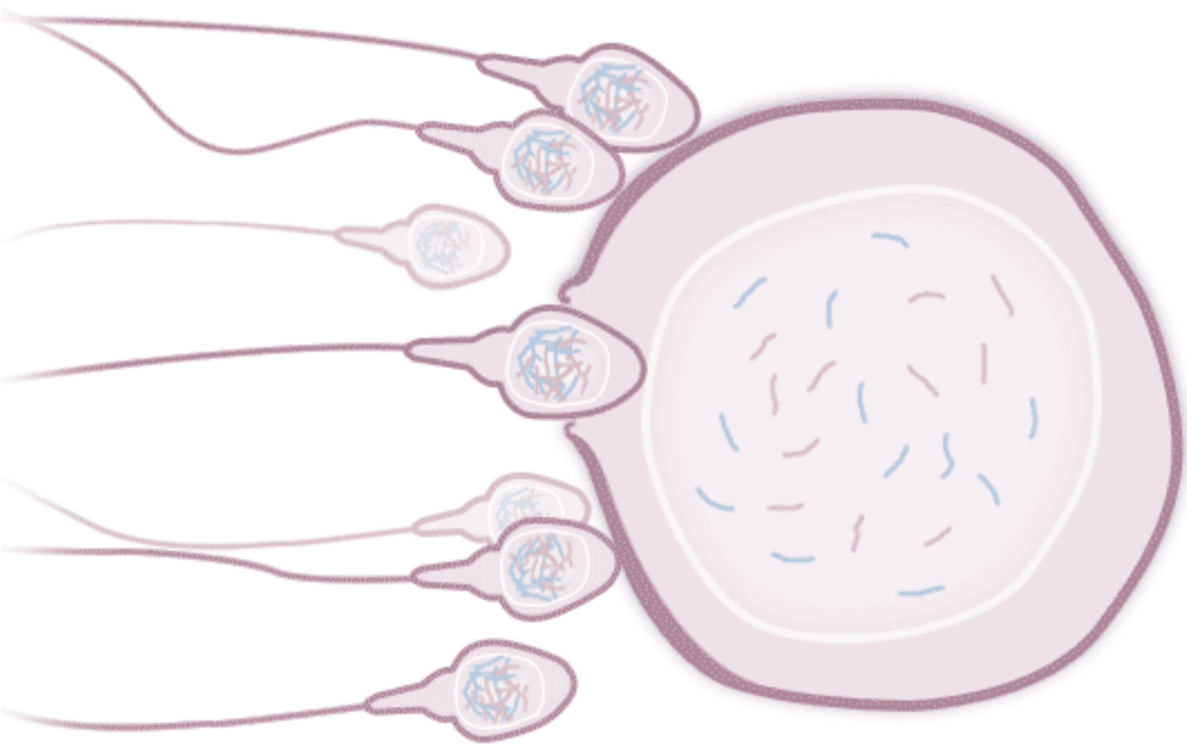

DU TILLFRÅGAS HÄRMED OM ATT DELTA I EN UNDERSÖKNING OM INFERTILITET

Syftet med detta projekt är att få mer kunskap om vilka förändringar i livsstil infertila par har gjort för att förbättra chanserna till graviditet samt kvinnans förhållande till sexualitet, psykisk hälsa och välbefinnande vid behandlingsstarten och efter att behandlingen avslutats antingen genom att hon blivit gravid eller därför att man inte har mer att erbjuda. Studien avser öka kunskapen om behovet av rådgivning till infertila par med olika kulturell och socioekonomisk bakgrund. Målet är att fylla de kunskapsluckor som finns idag avseende graviditetsplanering och livsstilsförändringar i samband med infertilitet och graviditet hos kvinnor. Resultaten från studien kan ligga till grund för att utveckla evidensbaserad rådgivning gällande livsstilsfaktorer för par som söker sig till fertilitetsklinikerna, vilket skulle kunna bidra till bättre behandlingsresultat

Vi behöver dina kontaktuppgifter för att kunna återkomma med följdfrågor om hur du mår och hur behandlingen har gått, efter cirka två år. Följdfrågorna avser komplettera den första enkäten med förändringar i och påverkan på livsstil under och efter behandlingsperioden.

Ditt personnummer kommer även att samköras med Medicinska födelseregistret och läkemedelsregistret. Detta endast för att kunna få information om behandlingsresultat, eventuella komplikationer samt eventuell graviditetsutfall. Information kan även insamlas ur journaluppgifter gällande vilka behandlingar du har gått igenom och vad är det för resultat. Dina uppgifter kommer inte att användas i något annat syfte än för detta forskningsprojekt. Resultaten analyseras på gruppnivå och ingen enskild individ kan identifieras. Dina svar kommer inte att kunna kopplas till din person. Ansvarig myndighet för registret är Institutionen för folkhälso- och vårdvetenskap, Uppsala universitet respektive Västmanland läns landsting.

Dina kontaktuppgifter sparas via en kodnyckel och hanteras i enlighet med personuppgiftslagen (PuL). Detta innebär att alla personuppgifter behandlas på ett sådant sätt att den personliga integriteten inte kränks. Allt insamlat material: kodnyckel, enkäter och datafiler handhas bara av projektgruppen och det kommer att förvaras i låsta utrymmen på institutionen för folkhälso- och vårdvetenskap.

Deltagandet i studien är frivilligt. Genom att fylla i ditt personnummer nedan godkänner du att delta i projektet. Tveka inte att ringa eller maila oss om du har frågor, kontaktuppgifterna hittar du nedan. Samtyckesblanketten förvarar vi åtskilt från din enkät. Din enkät får bara ett kodnummer.

Ansvariga samt kontaktpersoner
för undersökningen är:

UPPSALA
UNIVERSITET

Tanja Tydén - Leg. barnmorska, med. dr., professor vid Institutionen för folkhälso- och vårdvetenskap, Uppsala universitet
Tel. 018-471 66 46 • E-post: tanja.tyden@pubcare.uu.se

Kjell Wånggren - Överläkare, med. dr., specialist i obstetrik och gynekologi, Reproduktionscentrum och Institutionen för kvinnors och barns hälsa, Uppsala universitet
Tel. 018-611 57 52• E-post: kjell.wanggren@kbh.uu.se

Anna Berglund – Överläkare, med. dr., specialist i obstetrik och gynekologi, Nationellt centrum för kvinnofrid, Uppsala universitet.
Tel. 018-611 00 00 • E-post: anna.berglund@kbh.uu.se

Lana Salih – Läkare, specialist i obstetrik och gynekologi, Reproduktionscentrum, Kvinnokliniken, Akademiska sjukhuset, Uppsala
Tel. 018-611 00 00. • E-post: lana.salih@kbh.uu.se

Samtyckesblankett

Jag samtycker till att delta i studien om livsstil, hälsa och barnlöshet. Jag har blivit informerad om studien via skriftlig information och fått tillräcklig med tid på mig att överväga mitt deltagande. Jag vet att jag när som helst kan avsluta mitt deltagande i studien.

Jag samtycker till att mina personuppgifter behandlas som beskrivet i patientinformationen och är medveten om att mitt deltagande är helt frivilligt.

Jag får en kopia av patientinformationen och samtyckesblanketten.

Som tack för min medverkan erhåller jag en SF biobiljett.

Personnummer: ☐☐☐☐☐☐-☐☐☐☐

Namn:……………………………………………………………………………………

Adress:…………………………………………………………………………………

…………………………………………………………………………………………

Mobilnr :…………………………………… Telenr:……………………………………

E-post: …………………………………………………………………………………

Jag föredrar att bli kontaktad/få påminnelser via:

☐ Sms ☐ E-post ☐ Post ☐ Telefonsamtal

Obs: Denna sida kommer att avlägsnas från enkäten. Dina personuppgifter kommer att förvaras separat.

UPPSALA UNIVERSITET

ALLMÄNNA FRÅGOR

1. Ange dagens datum: År:……………….. Månad:……………….. Dag:………………..

2. Hur gammal är du? ……………….. år gammal

3. Vilken/vilka är din(a) nuvarande sysselsättning(ar)? (*Kryssa för alla alternativ som stämmer*)

	1-24%	25%	26-49%	50%	51-74%	75%	76-99%	100%
☐ Fast anställd/tillsvidare	☐	☐	☐	☐	☐	☐	☐	☐
☐ Egen företagare	☐	☐	☐	☐	☐	☐	☐	☐
☐ Tillfälligt anställning/vikariat	☐	☐	☐	☐	☐	☐	☐	☐
☐ Studerande	☐	☐	☐	☐	☐	☐	☐	☐
☐ Föräldraledig	☐	☐	☐	☐	☐	☐	☐	☐
☐ Sjukskriven	☐	☐	☐	☐	☐	☐	☐	☐
☐ Arbetslös	☐	☐	☐	☐	☐	☐	☐	☐
☐ Hemmafru/sköter hushållet	☐	☐	☐	☐	☐	☐	☐	☐
☐ Förtids- /sjukpensionerad	☐	☐	☐	☐	☐	☐	☐	☐

4. Vilken är den **högsta** utbildning du har **avslutat**?

   ☐ Har inte avslutat någon utbildning

   ☐ Grundskola

   ☐ Gymnasieskola

   ☐ Yrkeshögskola eller kvalificerad yrkesutbildning

   ☐ Universitet eller högskola – 2,5 år eller kortare

   ☐ Universitet eller högskola – 3,5 år

   ☐ Universitet eller högskola – Forskarutbildning

5. Hur stor är månadsinkomsten före skatt i ditt hushåll?
   (Med inkomst menas lön, a-kassa, sjuk-/föräldrapenning, pension, underhållsstöd, barnbidrag, efterlevandestöd, socialförsäkringsförmåner och andra ersättningar.)

   ………………… kr/månad

6. Hur lång är du? ………………..cm

7. Hur mycket väger du idag? ………………..kg

8. Hur mycket vägde du **för ett år sedan?** ………………..kg

9. a. I vilket land är du född? ☐ Sverige ☐ Annat land: ....

b. Om du är född i ett annat land än Sverige, hur gammal var du när du kom till Sverige? ....år ☐ Mindre än 1 år gammal

c. Är du adopterad? ☐ Ja ☐ Nej

10. I vilket land är dina föräldrar födda?

a. Mamma är född i: ☐ Sverige ☐ Annat land: ....

☐ Vet ej

b. Pappa är född i: ☐ Sverige ☐ Annat land: ....

☐ Vet ej

11. Hur bra kan du läsa och förstå skriven svenska?

Mycket bra	Ganska bra	Varken bra eller dåligt	Ganska dåligt	Mycket dåligt
☐	☐	☐	☐	☐

12. Hur stor betydelse har religion i ditt liv?

Mycket stor betydelse	Ganska stor betydelse	Varken stor eller liten betydelse	Ganska liten betydelse	Mycket liten betydelse
☐	☐	☐	☐	☐

NÅGRA FRÅGOR OM DIN NUVARANDE PARTNER

13. Vilket kön har din nuvarande partner? ☐ Man ☐ Kvinna

14. När är din partner född? År: 19 …………..

15. Hur länge har du och din nuvarande partner haft ett förhållande? (*Kryssa för det alternativ som stämmer bäst.*)

mindre än 1 år	1 år	2 år	3 år	4 år	5 år	6 år	7 år	8 år eller mer
☐	☐	☐	☐	☐	☐	☐	☐	☐

16. Vilken är din relation till din nuvarande partner? *(Kryssa för alla alternativ som stämmer)*

☐ Vi har ett förhållande men bor inte tillsammans

☐ Vi är sambo/bor tillsammans

☐ Vi är förlovade

☐ Vi är gifta

17. a. Har **din nuvarande partner** några egna biologiska barn, som du inte är biologisk förälder till?

☐ Nej ☐ Ja, 1 barn ☐ Ja, 2 barn ☐ Ja, 3 barn ☐ Ja, 4 barn eller fler

b. Har **du** några egna biologiska barn, som din nuvarande partner inte är biologisk förälder till?

☐ Nej ☐ Ja, 1 barn ☐ Ja, 2 barn ☐ Ja, 3 barn ☐ Ja, 4 barn eller fler

18. I vilket land är din partner född? ☐ Sverige ☐ Annat land: ……………………………………

☐ Vet ej

19. Vilken är den **högsta** utbildning din nuvarande partner har **avslutat**?

☐ Har inte avslutat någon utbildning

☐ Grundskola

☐ Gymnasieskola

☐ Yrkeshögskola eller kvalificerad yrkesutbildning

☐ Universitet eller högskola – 2,5 år eller kortare

☐ Universitet eller högskola – 3,5 år

☐ Universitet eller högskola – Forskarutbildning

20. Röker din nuvarande partner? ☐ Nej ☐ Ja, dagligen ☐ Ja, men inte dagligen

21. För följande sju frågor, markera med ett kryss det svar som bäst motsvarar din syn på din nuvarande parrelation.

a. Hur bra svarar din partner an mot dina behov i allmänhet?	Inte alls ☐	☐	Lika bra ☐	☐	Fullständigt ☐
b. Hur tillfredsställd är du i allmänhet med relationen till din partner?	Inte alls ☐	☐	Lika bra ☐	☐	Fullständigt ☐
c. Hur bra är din relation till din partner i allmänhet om du jämför med de flesta andras parrelationer?	Mycket sämre ☐	☐	Lika bra ☐	☐	Mycket bättre ☐
d. Hur ofta önskar du att du inte hade blivit tillsammans med din partner?	Aldrig ☐	☐	Lika bra ☐	☐	Alltid ☐
e. I vilken utsträckning har relationen till din partner motsvarat dina ursprungliga förväntningar?	Inte alls ☐	☐	Lika bra ☐	☐	Fullständigt ☐
f. Hur mycket tycker du om din partner?	Ytterst lite ☐	☐	Lika bra ☐	☐	Väldigt mycket ☐
g. Hur många problem har ni i er relation?	Ytterst få ☐	☐	Lika bra ☐	☐	Väldigt många ☐

DIN LIVSSTIL FÖR NÄRVARANDE

22. Vilken typ av kost äter du **för närvarande**? *(Kryssa för det alternativ som stämmer **bäst** för dig)*

- ☐ Allsidigt sammansatt kost (Du äter det mesta och undviker inte någon specifik livsmedelsgrupp)
- ☐ Vegetarisk kost
- ☐ Vegankost
- ☐ Undviker vissa livsmedel på grund av födoämnesöverkänslighet
- ☐ Undviker vissa livsmedel av religiösa skäl
- ☐ LCHF-kost (Du äter mat med få kolhydrater men mycket fett)
- ☐ GI-kost (Du äter mat med lågt GI-värde/glykemiskt index)
- ☐ Annan kost, beskriv

____

____

____

____

23. a. Tar du tillskott av folsyra **för närvarande?**

☐ Ja ☐ Nej → *Fortsätt till fråga 24*

b. Om du tar tillskott av folsyra **för närvarande, när** började du ta detta?

0-2 veckor sedan	3-4 veckor sedan	1-2 månader sedan	3-4 månader sedan	5-6 månader sedan	7-12 månader sedan	1-2 år sedan	3-4 år sedan	Mer än 4 år sedan
☐	☐	☐	☐	☐	☐	☐	☐	☐

c. Under den tiden du har tagit tillskott av folsyra, **hur ofta** har du tagit detta?

Minst 5 gånger/vecka	3 - 4 gånger/vecka	1 - 2 gånger/vecka	2 - 3 gånger/månad	1 gång/månad	1 - 6 gånger/år
☐	☐	☐	☐	☐	☐

24. a. Tar du tillskott av multivitamin för kvinnor **för närvarande?**

☐ Ja ☐ Nej → *Fortsätt till fråga 25*

b. Om du har tar tillskott av multivitamin för kvinnor **för närvarande, när** började du ta detta? *(Svara i veckor, månader,* ***eller*** *år.)*

0-2 veckor sedan	3-4 veckor sedan	1-2 månader sedan	3-4 månader sedan	5-6 månader sedan	7-12 månader sedan	1-2 år sedan	3-4 år sedan	Mer än 4 år sedan
☐	☐	☐	☐	☐	☐	☐	☐	☐

c. Under den tiden du har tagit tillskott av multivitamin för kvinnor, **hur ofta** har du tagit detta?

Minst 5 gånger/vecka	3 - 4 gånger/vecka	1 - 2 gånger/vecka	2 - 3 gånger/månad	1 gång/månad	1 - 6 gånger/år
☐	☐	☐	☐	☐	☐

25. Använder du något receptfritt läkemedel eller naturläkemedel? *(Kryssa för alla alternativ som stämmer)*

☐ Näsdroppar

☐ Medel mot förkylning

☐ Vätskedrivande medel

☐ Smärtstillande eller antiinflammatoriska medel

☐ Lugnande medel

☐ Sömnmedel

☐ Medel mot allergi

☐ Rökavvänjningsmedel (t.ex. nikotintuggummi)

☐ Annat receptfritt läkemedel

☐ Annat naturläkemedel

☐ Nej, använde bara receptbelagda läkemedel

☐ Nej, använde inga läkemedel eller naturläkemedel

26. Hur mycket kaffe dricker du i genomsnitt *(1kopp=1,5dl)*? (*Svara i antal koppar/dag)*

Färre än 1 kopp/dag	1 kopp/d.	2 koppar/d.	3 koppar/d.	4 koppar/d.	5 koppar/d.	6 koppar/d.	7 koppar/d.	8 eller fler koppar/d.
☐	☐	☐	☐	☐	☐	☐	☐	☐

eller ☐ Dricker inte kaffe

Här följer några frågor om dina alkoholvanor. I dessa frågor används uttrycket "standardglas alkohol".

Ett STANDARDGLAS ALKOHOL motsvarar följande:

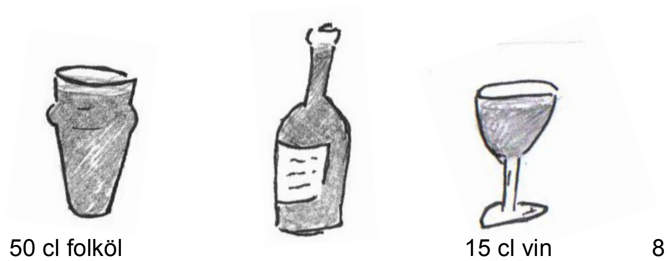

50 cl folköl 33 cl starköl/ starkcider 15 cl vin 8 cl starkvin 4 cl sprit

27. a. Dricker du alkohol **för närvarande**? *(Kryssa för det alternativ som stämmer **bäst** för dig)*

☐ Ja, jag dricker varje vecka.

☐ Ja, men inte varje vecka.

☐ Nej, jag slutade dricka alkohol **när** jag inledde mitt nuvarande graviditetsförsök

☐ Nej, jag hade slutat dricka alkohol **innan** jag inledde mitt nuvarande graviditetsförsök

☐ Nej, jag har aldrig druckit alkohol

b. Hur många standardglas alkohol/vecka dricker du i genomsnitt ***för närvarande?***

Mindre än 1 glas/v.	1 glas/v.	2 glas/v.	3 glas/v.	4 glas/v.	5 glas/v.	6 glas/v.	7 glas/v.	8 eller fler glas/v.
☐	☐	☐	☐	☐	☐	☐	☐	☐

c. Hur många gånger under det senaste **halvåret** har du druckit **fem standardglas eller mer vid ett tillfälle**?

0 ggr	1 gång	2 ggr	3 ggr	4 ggr	5 ggr	6 ggr	7 ggr	8 eller fler gånger
☐	☐	☐	☐	☐	☐	☐	☐	☐

28. a. Röker du **för närvarande**? *(Kryssa för det alternativ som stämmer **bäst** för dig)*

☐ Ja, jag röker varje dag.

☐ Ja, men inte varje dag.

☐ Nej, jag har slutat röka → *Fortsätt till fråga 29*

☐ Nej, jag har aldrig rökt → *Fortsätt till fråga 29*

b. Hur många cigaretter/dag röker du i genomsnitt?

färre än 1 cigg/dag	1 cigg/dag	2 - 3 cigg/dag	4 - 5 cigg/dag	6 - 9 cigg/dag	10 - 14 cigg/dag	15 - 20 cigg/dag	21 - 30 cigg/dag	31 eller fler cigg/d
☐	☐	☐	☐	☐	☐	☐	☐	☐

29. a. Snusar du för närvarande?

☐ Ja, jag snusar varje dag

☐ Ja, men inte varje dag

☐ Nej, jag hade slutat snusa sedan tidigare → *Fortsätt till fråga 30*

☐ Nej, jag har aldrig snusat → *Fortsätt till fråga 30*

b. Hur många portioner snus/dag använder du i genomsnitt **för närvarande**?

färre än 1 port/dag	1 port/dag	2 - 3 port/dag	4 - 5 port/dag	6 - 9 port/dag	10 - 14 port/dag	15 - 20 port/dag	21 - 30 port/dag	31 eller fler port/d.
☐	☐	☐	☐	☐	☐	☐	☐	☐

30. Använder du nikotinersättningsmedel *(t.ex. nikotintuggummi eller nikotinplåster)* **för närvarande**?

☐ Ja ☐ Nej

31. Hur många timmar har du i genomsnitt sovit per dygn en normal vardag den **senaste månaden**?

5 eller färre tim.	6 tim.	7 tim.	8 tim.	9 tim.	10 tim.	11 tim.	12 tim.	13 eller fler tim.
☐	☐	☐	☐	☐	☐	☐	☐	☐

32. Hur många timmar/vecka ägnade du dig åt någon fysisk aktivitet den senaste månaden? (Avrunda till hela antal timmar.)

0 tim/v	1 tim/v	2 tim/v	3 tim/v	4 tim/v	5 tim/v	6 - 10 tim/v	11 – 20 tim/v	21 eller fler tim/v
☐	☐	☐	☐	☐	☐	☐	☐	☐

33. Hur många gånger i veckan har du i genomsnitt vaginalt samlag? (Ange i hela antal gånger)

Färre än 1 gång/v.	1 gång/v.	2 ggr/v.	3 ggr/v.	4 ggr/v.	5 ggr/v.	6 ggr/v.	7 ggr/v.	8 eller fler ggr/v.
☐	☐	☐	☐	☐	☐	☐	☐	☐

34. Hur nöjd eller missnöjd är du med ditt sexliv?

Mycket missnöjd	Ganska missnöjd	Varken nöjd eller missnöjd	Ganska nöjd	Mycket nöjd
☐	☐	☐	☐	☐

35. När var din senaste mens första dag? *(Om du inte minns exakt dag, ange bara år och månad)*

År 20……… Månad: …………. Dag: ………..

36. Hur stark menstruationssmärta hade du vid din senaste mens?

Mycket stark smärta	Ganska stark smärta	Varken stark eller svag smärta	Ganska svag smärta	Mycket svag smärta
☐	☐	☐	☐	☐

37. Hur gammal var du när du fick din första menstruation? ……………….. år gammal

38. Hur lång är vanligtvis din **menstruationscykel**? *(Med **menstruationscykel** menas tiden mellan första dagen i en menstruation och första dagen i nästa menstruation)*

20 dagar eller färre	21 – 25 dagar	26 dagar	27 dagar	28 dagar	29 dagar	30 dagar	31 – 35 dagar	36 dagar eller fler
☐	☐	☐	☐	☐	☐	☐	☐	☐

39. Har du eller har du tidigare haft någon av följande diagnoser/sjukdomar? *(Kryssa för alla alternativ som stämmer)*

☐ ADHD	☐ Hjärtsjukdom
☐ Allergi/intolerans mot någon mat	☐ Hypertoni/högt blodtryck
☐ Astma	☐ Klamydia
☐ Bipolär sjukdom	☐ Ledvärk
☐ Depression	☐ Njursjukdom
☐ Diabetes	☐ PCOS *(polycystiskt ovariesyndrom)*
☐ Endometrios	☐ Ryggvärk
☐ Epilepsi	☐ Ångest
☐ Gynekologisk cellförändring	☐ Ätstörning *(t.ex. bulimi eller anorexi)*
☐ Sköldkörtelsjukdom *(t.ex. hypotyreos eller hypertyreos)*	

40. Har du haft någon annan sjukdom/diagnos än de som frågades efter i föregående fråga?

☐ Nej ☐ Ja, jag har haft följande sjukdom(ar)/diagnos(er): ………………………………………

………

41. Har du någon gång genomgått en bukoperation? ☐ Ja ☐ Nej

42. Hur bedömer du att ditt allmänna hälsotillstånd är **för närvarande**?

Mycket bra	Ganska bra	Varken bra eller dåligt	Ganska dåligt	Mycket dåligt
☐	☐	☐	☐	☐

EVENTUELLA TIDIGARE GRAVIDITETER

43. a. Har du varit gravid tidigare *(räkna även in missfall och aborter)?*

☐ Ja ☐ Nej → *Fortsätt till fråga 44*

b. Hur många gånger har du varit gravid *(räkna även in missfall och aborter)*?

1 gång	2 ggr	3 ggr	4 ggr	5 ggr	6 ggr	7 ggr	8 ggr	9 eller fler ggr
☐	☐	☐	☐	☐	☐	☐	☐	☐

c. Hur gammal var du när du blev gravid för första gången *(räkna även in missfall och aborter)*?

……………….. år

d. Hur många gånger har du råkat ut för missfall i månad 0-6?

☐ 0 ggr ☐ 1 gång ☐ 2 ggr ☐ 3 ggr ☐ 4 ggr ☐ 5 ggr ☐ 6 eller fler ggr

e. Hur många gånger har du genomgått abort?

☐ 0 ggr ☐ 1 gång ☐ 2 ggr ☐ 3 ggr ☐ 4 ggr ☐ 5 ggr ☐ 6 eller fler ggr

f. Hur många gånger har du haft utomkvedshavandeskap?

☐ 0 ggr ☐ 1 gång ☐ 2 ggr ☐ 3 ggr ☐ 4 ggr ☐ 5 ggr ☐ 6 eller fler ggr

44. a. Har du fött barn tidigare? ☐ Ja ☐ Nej → *Fortsätt till fråga 45*

	0	1	2	3	4	5 eller fler
b. Om ja, hur många?		☐	☐	☐	☐	☐
c. Hur många föddes levande?	☐	☐	☐	☐	☐	☐
d. Hur många föddes mer än 3 månader för tidigt?	☐	☐	☐	☐	☐	☐
e. Hur många hade en medfödd missbildning?	☐	☐	☐	☐	☐	☐
f. Hur många biologiska barn har du idag?	☐	☐	☐	☐	☐	☐

OMSTÄNDIGHETER KRING DITT NUVARANDE GRAVIDITETSFÖRSÖK

Här nedan följer några frågor som berör omständigheterna kring ditt/ert nuvarande graviditetsförsök. Med detta avses tiden från att ni inledde ett aktivt försök att bli gravida fram till och med dagens datum.

45. Sedan när har du och din nuvarande partner aktivt försökt att bli gravida?

År: ……………….. Månad: ……………………

46. Vem tog **initiativet** till beslutet att du skulle försöka bli gravid?

☐ Bara jag ☐ Mest jag

☐ Jag och min partner lika mycket

☐ Bara min partner ☐ Mest min partner

☐ Annan/andra, beskriv: ……………………………………………………………………………

47. Satte du/ni upp en **tidsram** inom vilken du/ni förväntade att du skulle bli gravid?

☐ Ja, jag/vi förväntade att jag skulle bli gravid inom …………….. veckor / ……………...månad(er)

☐ Nej

48. Har du använt dig av ägglossningstest under tiden som ditt nuvarande graviditetsförsök har pågått?

☐ Nej ☐ Ja, jag har använt ………….. st ägglossningstest

49. Har du under ditt nuvarande graviditetsförsök övervägt att adoptera ett barn?

Ja, mycket	Ja, lite	Varken ja eller nej	Nej, inte direkt	Nej, inte alls
☐	☐	☐	☐	☐

50. Har du fått personlig rådgivning om infertilitet eller ditt nuvarande graviditetsförsök av någon av följande yrkeskategorier? *(Kryssa för allt som stämmer)*

☐ Husläkare ☐ Specialistläkare ☐ Barnmorska

☐ Sjuksköterska ☐ Dietist ☐ Kurator

☐ Annan: ……………………………………………………………………………………………...

☐ Nej, har inte fått personlig rådgivning omkring infertilitet eller mitt nuvarande graviditetsförsök

51. Har du, under tiden från att du inledde ditt nuvarande graviditetsförsök, gjort något för att förbättra din hälsa som förberedelse för graviditet? *(Kryssa för allt som stämmer)*

- ☐ Tagit folsyra
- ☐ Slutat röka
- ☐ Dragit ner på rökningen
- ☐ Slutat snusa
- ☐ Dragit ner på snusningen
- ☐ Slutat dricka alkohol
- ☐ Dragit ner på alkoholintaget
- ☐ Slutat dricka kaffe
- ☐ Dragit ner på kaffeintaget
- ☐ Motionerat mer
- ☐ Motionerat mindre
- ☐ Ändrat din medicinering
- ☐ Ätit mer hälsosamt, beskriv: ……………………………………………………………………………
- ☐ Sökt medicinsk rådgivning/hälsorådgivning
- ☐ Vidtagit andra åtgärder, beskriv: ……………………………………………………………………

eller

- ☐ Jag har inte gjort något av ovanstående

52. Var har du och/eller din partner sökt information om infertilitet eller ditt nuvarande graviditetsförsök? *(Kryssa för allt som stämmer)*

- ☐ Mödravårdscentral / Barnmorskemottagning
- ☐ Annan hälso- och sjukvårdsinstans
- ☐ Familj, vänner och bekanta
- ☐ Livsmedelsverket
- ☐ Statens Folkhälsoinstitut
- ☐ Internetforum *(internetsidor: gravid, familjeliv, growingpeople, alltförföräldrar)*
- ☐ Vårdsidor på internet *(t.ex. 1177, vårdguiden)*
- ☐ Andra internetkällor:.………………………………………………………………………………...
- ☐ Tidningar
- ☐ Böcker
- ☐ Annat: ………………………………………………………………………….…………………..
- ☐ Har inte sökt information om infertilitet eller mitt nuvarande graviditetsförsök

53. Markera med ett kryss det svar som bäst motsvarar hur du har känt det **den senaste veckan**.

a.	Jag känner mig spänd och nervös	Väldigt ofta ☐	Ofta ☐	Sällan ☐	Inte alls ☐
b.	Jag har en känsla av att något hemskt kommer att hända	Mycket klart och obehagligt ☐	Inte så starkt nu ☐	Betydligt svagare nu ☐	Inte alls ☐
c.	Jag bekymrar mig över saker	Väldigt ofta ☐	Ofta ☐	Sällan ☐	Inte alls ☐
d.	Jag kan sitta stilla och känna mig avslappnad	Väldigt ofta ☐	Ofta ☐	Sällan ☐	Inte alls ☐
e.	Jag känner mig orolig, som om jag hade fjärilar i magen	Väldigt ofta ☐	Ofta ☐	Sällan ☐	Inte alls ☐
f.	Jag känner mig rastlös	Väldigt ofta ☐	Ofta ☐	Sällan ☐	Inte alls ☐
g.	Jag får plötsliga panikkänslor	Väldigt ofta ☐	Ofta ☐	Sällan ☐	Inte alls ☐

54. För följande 10 frågor, kryssa för det alternativ som bäst beskriver hur du har känt det **den senaste veckan**.	
a. Jag har kunnat skratta och se tillvaron från den ljusa sidan<br>☐ Lika bra som vanligt<br>☐ Nästan lika bra som vanligt<br>☐ Mycket mindre än vanligt<br>☐ Inte alls	f. Det har kört ihop sig för mig och blivit för mycket<br>☐ Ja, mesta tiden har jag inte kunnat ta itu med något alls<br>☐ Ja, ibland har jag inte kunnat ta itu med saker lika bra som vanligt<br>☐ Nej, för det mesta har jag kunnat ta itu med saker ganska bra<br>☐ Nej, jag har kunnat ta itu med saker precis som vanligt
b. Jag har glatt mig åt saker som skall hända<br>☐ Lika mycket som vanligt<br>☐ Något mindre än vanligt<br>☐ Mycket mindre än vanligt<br>☐ Inte alls	g. Jag har känt mig så olycklig att jag har haft svårt att sova<br>☐ Ja, för det mesta<br>☐ Ja, ibland<br>☐ Nej, sällan<br>☐ Nej, aldrig
c. Jag har lagt skulden på mig själv onödigt mycket när något gått snett<br>☐ Ja, för det mesta<br>☐ Ja, ibland<br>☐ Inte så ofta<br>☐ Nej, aldrig	h. Jag har känt mig ledsen och nere<br>☐ Ja, för det mesta<br>☐ Ja, rätt ofta<br>☐ Nej, sällan<br>☐ Nej, aldrig
d. Jag har känt mig rädd och orolig utan egentlig anledning<br>☐ Nej, inte alls<br>☐ Ganska sällan<br>☐ Ja, ibland<br>☐ Ja, mycket ofta	i. Jag har känt mig så olycklig att jag gråtit<br>☐ Ja, nästan jämt<br>☐ Ja, ganska ofta<br>☐ Bara någon gång<br>☐ Aldrig
e. Jag har känt mig skrämd eller panikslagen utan speciell anledning<br>☐ Ja, mycket ofta<br>☐ Ja, ibland<br>☐ Nej, ganska sällan<br>☐ Nej, inte alls	j. Tankar på att göra mig själv illa har förekommit<br>☐ Ja, rätt ofta<br>☐ Ja, då och då<br>☐ Ganska sällan<br>☐ Aldrig

55.Hur ofta har Du under **den senaste månaden** …

		Aldrig	Sällan	Ibland	Ganska ofta	Mycket ofta
a.	… blivit upprörd över något som skett helt oväntat?	☐	☐	☐	☐	☐
b.	… känt att Du inte haft kontroll över de viktiga faktorerna i ditt liv?	☐	☐	☐	☐	☐
c.	… känt Dig nervös och stressad?	☐	☐	☐	☐	☐
d.	… känt Dig säker på Din förmåga att hantera personliga problem?	☐	☐	☐	☐	☐
e.	… tyckt att saker och ting har utvecklat sig som Du velat?	☐	☐	☐	☐	☐
f.	… känt att Du inte kunde hantera allt som måste göras?	☐	☐	☐	☐	☐
g.	… känt att Du har haft kontroll över irriterande moment i Ditt liv?	☐	☐	☐	☐	☐
h.	… känt att Du har haft kontroll över saker och ting?	☐	☐	☐	☐	☐
i.	… blivit arg över saker som har hänt och som låg utanför Din kontroll?	☐	☐	☐	☐	☐
j.	… känt att problemen blivit så många att Du inte kunnat bemästra dem?	☐	☐	☐	☐	☐

GRAVIDITET I ALLMÄNHET

Här följer några frågor om vad du tror om förmågan att bli gravid. OBS! Detta är inte ett kunskapstest – vi vill bara veta vad du tror.

56. Hur stor tror du chansen i genomsnitt är att en kvinna i din ålder blir gravid, om hon har oskyddat vaginalt samlag **precis vid ägglossningen**?

    .... procents chans per ägglossningstillfälle

57. Hur stor tror du chansen i genomsnitt är, per försök, att en kvinna i din ålder blir gravid **med hjälp av provrörsbefruktning**?

    .... procents chans per försök

58. Vid vilken ålder tror du att kvinnor är som mest fruktsamma? .... år

59. Vid vilken ålder tror du att en **viss** nedgång i en kvinnas förmåga att bli gravid börjar? .... år

60. Vid vilken ålder tror du att en **påtaglig** nedgång i en kvinnas förmåga att bli gravid börjar? .... år

61. Har du upplevt följande aspekter som stödjande eller som hindrande för att få/ha barn**?**

	Mycket stödjande	Ganska stödjande	Varken stödjande eller hindande	Ganska hindrande	Mycket hindrande	Ej aktuellt
Din partner	☐	☐	☐	☐	☐	☐
Din familj	☐	☐	☐	☐	☐	☐
Dina vänner	☐	☐	☐	☐	☐	☐
Din ålder	☐	☐	☐	☐	☐	☐
Din mognad	☐	☐	☐	☐	☐	☐
Din boende situation	☐	☐	☐	☐	☐	☐
Din karriär	☐	☐	☐	☐	☐	☐
Dina anställningsförhållanden	☐	☐	☐	☐	☐	☐
Din ekonomi	☐	☐	☐	☐	☐	☐
Vilken föräldrapenning du har rätt till	☐	☐	☐	☐	☐	☐

NÅGRA FRÅGOR OM DIN UPPVÄXT

Här följer några frågor om din uppväxt. Om frågorna väcker ett behov av att få prata med någon utomstående kan du vända dig till din läkare som hänvisar dig till den hjälp du behöver.

62. Under dina första 18 levnadsår:

	Ja	Nej
a. Levde du tillsammans med någon som hade problematiska alkoholvanor eller var alkoholist?	☐	☐
b. Levde du tillsammans med någon som använde droger?	☐	☐
c. Var dina föräldrar någonsin separerade eller skilda?	☐	☐
d. Var någon i ditt hem deprimerad eller psykiskt sjuk?	☐	☐
e. Försökte någon i ditt hem begå självmord?	☐	☐
f. Var någon i ditt hem någonsin satt i fängelse?	☐	☐

63. Under dina första 18 levnadsår:

	Aldrig	En, två gånger	Ibland	Ofta	Mycket ofta
a. Hur ofta kände du att ingen i din familj älskade dig eller tyckte att du var viktig eller speciell?	☐	☐	☐	☐	☐
b. Hur ofta kände du att i din familj brydde man sig inte om varandra, stod inte varandra känslomässigt nära och stöttade inte varandra?	☐	☐	☐	☐	☐
c. Hur ofta hade du inte tillräckligt att äta, var tvungen att ha smutsiga kläder på dig och upplevde att ingen tog hand om dig?	☐	☐	☐	☐	☐
d. Hur ofta var dina föräldrar för berusade eller drogade för att ta hand om dig eller ta dig till läkare om du behövde det?	☐	☐	☐	☐	☐

64. Ibland förekommer det att föräldrar eller andra vuxna skadar barn. Under din uppväxt, det vill säga under dina första 18 levnadsår, hur ofta förekom det att en förälder, styvförälder eller vuxen boende i ditt hem:

	Aldrig	En, två gånger	Ibland	Ofta	Mycket ofta
a. Svor åt dig, förolämpade dig eller kränkte dig?	☐	☐	☐	☐	☐
b. Hotade att slå dig eller kasta något på dig, men gjorde det inte?	☐	☐	☐	☐	☐
c. Agerade på ett sätt som gjorde dig rädd att du kanske skulle bli fysiskt skadad?	☐	☐	☐	☐	☐
d. Faktiskt knuffade, grep tag, föste, slog eller kastade något på dig?	☐	☐	☐	☐	☐
e. Slog dig så hårt att du fick märken eller blev skadad?	☐	☐	☐	☐	☐

65. Ibland förekommer fysiskt våld mellan föräldrar. Under dina första 18 levnadsår hur ofta gjorde din pappa (eller styvpappa) eller din mammas pojkvän/partner någon/några av dessa **saker mot din mamma (eller styvmamma)**?

	Aldrig	En, två gånger	Ibland	Ofta	Mycket ofta
a. Knuffade, grep tag, slog eller kastade något på henne?	☐	☐	☐	☐	☐
b. Sparkade, bet eller slog henne med knuten näve eller med något hårt?	☐	☐	☐	☐	☐
c. Slog henne upprepat under åtminstone några minuter?	☐	☐	☐	☐	☐
d. Hotade henne med kniv eller skjutvapen, eller använde kniv eller skjutvapen för att skada henne?	☐	☐	☐	☐	☐

66. Ibland förekommer fysiskt våld mellan föräldrar. Under dina första 18 levnadsår hur ofta gjorde din mamma (eller styvmamma) eller din pappas flickvän/partner någon/några av dessa **saker mot din pappa (eller styvpappa)**?

	Aldrig	En, två gånger	Ibland	Ofta	Mycket ofta
a. Knuffade, grep tag, slog eller kastade något på honom?	☐	☐	☐	☐	☐
b. Sparkade, bet eller slog honom med knuten näve eller med något hårt?	☐	☐	☐	☐	☐
c. Slog honom upprepat under åtminstone några minuter?	☐	☐	☐	☐	☐
d. Hotade honom med kniv eller skjutvapen, eller använde kniv eller skjutvapen för att skada honom?	☐	☐	☐	☐	☐

67. En del människor har under de första 18 levnadsåren av sin uppväxt sexuella upplevelser med en vuxen eller någon minst 5 år äldre än de själva. Dessa upplevelser kan ha involverat en släkting, vän till familjen eller främling. Under dina första 18 levnadsår har en vuxen eller äldre släkting, vän till familjen eller främling någon gång:

	Första gången detta inträffade, hur gammal var du?	Första gången, detta inträffade, skedde det mot din vilja?	Sista gången detta inträffade, hur gammal var du?	Ungefär hur många gånger hände detta dig?	Hur många människor gjorde detta med dig?	Vilket kön hade den/de som gjorde detta?
a. Berört eller smekt din kropp på ett sexuellt sätt?<br>☐ Ja ☐ Nej<br>Om "Ja" →	----------------- år	☐ Ja<br>☐ Nej	----------------- år	----------------- gånger	--------------- människor	☐ man<br>☐ kvinna<br>☐ bägge
b. Fick dig att beröra eller smeka sin kropp på ett sexuellt sätt?<br>☐ Ja ☐ Nej<br>Om "Ja" →	----------------- år	☐ Ja<br>☐ Nej	----------------- år	----------------- gånger	--------------- människor	☐ man<br>☐ kvinna<br>☐ bägge
c. Försökt att ha någon typ av sexuellt umgänge (oralt, analt eller vaginalt) med dig?<br>☐ Ja ☐ Nej<br>Om "Ja" →	----------------- år	☐ Ja<br>☐ Nej	----------------- år	----------------- gånger	--------------- människor	☐ man<br>☐ kvinna<br>☐ bägge
d. Faktiskt genomfört sexuellt umgänge (oralt, analt eller vaginalt) med dig?<br>☐ Ja ☐ Nej<br>Om "Ja" →	----------------- år	☐ Ja<br>☐ Nej	----------------- år	----------------- gånger	--------------- människor	☐ man<br>☐ kvinna<br>☐ bägge

68. Tyckte du att frågorna om din uppväxt (fråga 62-67) var obehagliga att svara på?

☐ Ja, jag vill inte svara

☐ Ja, men det känns okej att svara

☐ Nej, det är okej att svara

☐ Vet ej/Ingen åsikt

OM DITT BESÖK PÅ VÅR MOTTAGNING

69. Vad har du för förväntningar på ditt besök på vår mottagning?

☐ Få veta anledningen till att jag inte blivit gravid, och få behandling så fort som möjligt

☐ Få veta anledningen till att jag inte blivit gravid, men vill avvakta med behandlingen och fortsätta med egna försök ett tag till

☐ Annat, beskriv: ……………………………………………………………………………………………

………..

………..

………..

………..

………..

………..

………..

………..

………..

………..

………..

NÅGRA AVSLUTANDE FRÅGOR

70. Har din partner varit närvarande när du fyllt i denna enkät?

☐ Ja ☐ Nej

71. Har du tagit hjälp av någon när du fyllt i denna enkät?

☐ Nej, jag har inte tagit hjälp av någon

☐ Ja, av min make/partner

☐ Ja, av en tolk

☐ Ja, av läkaren/sjuksköterskan

☐ Ja, av någon annan. Ange vem/vilka: ………………………………………………………………...

KOMMENTARER TILL FRÅGEFORMULÄRET

Om du önskar göra tillägg eller kommentarer till detta frågeformulär, t.ex. om någon fråga var oklart formulerad, om du vill göra ett tillägg till någon fråga, eller om det var någon fråga som du saknade, så kan du göra det här. Alla kommentarer tas tacksamt emot.

……………………………………………………………………………

……………………………………………………………………………

……………………………………………………………………………

……………………………………………………………………………

……………………………………………………………………………

……………………………………………………………………………

……………………………………………………………………………

……………………………………………………………………………

……………………………………………………………………………

……………………………………………………………………………

……………………………………………………………………………

……………………………………………………………………………

……………………………………………………………………………

……………………………………………………………………………

……………………………………………………………………………

……………………………………………………………………………

……………………………………………………………………………

……………………………………………………………………………

……………………………………………………………………………

……………………………………………………………………………

……………………………………………………………………………

TACK FÖR DIN MEDVERKAN!

Om denna enkät har väckt ett behov av att få prata med någon utomstående kan du vända dig till din läkare som kan hänvisa dig till den hjälp du behöver.

Kodnummer:………………..
