# Supplementary material for: Investigating the effect of lifestyle risk factors upon number of aspirated and mature oocytes in in vitro fertilization cycles: Interaction with antral follicle count
Source: PLoS One. 2019 Aug 16;14(8):e0221015. doi: 10.1371/journal.pone.0221015 (PMC6697332; doi:10.1371/journal.pone.0221015)
Supplement: S2 Questionnaire — (PDF) [file pone.0221015.s005.pdf]

LIFESTYLE AND INFERTILITY

A STUDY ON FERTILITY

DEPARTMENT OF PUBLIC HEALTH AND CARING SCIENCES (IFV)

DEPARTMENT OF WOMEN'S AND CHILDREN'S HEALTH

UPPSALA UNIVERSITY

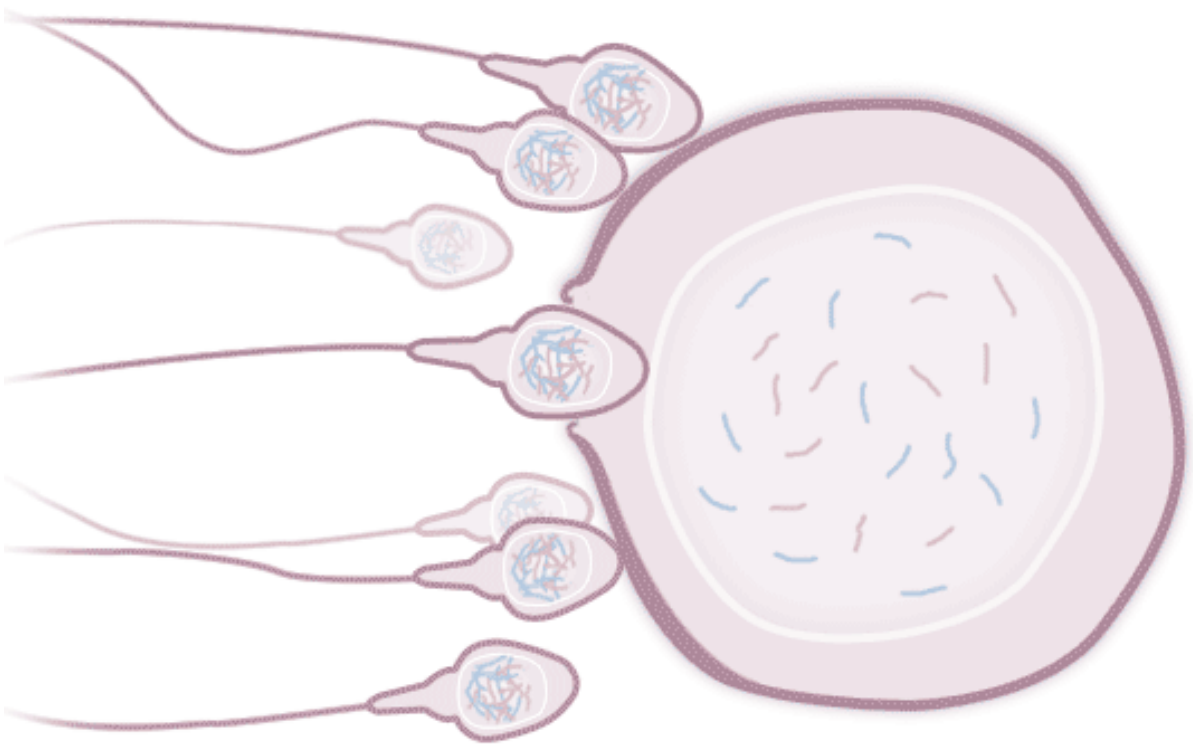

YOU ARE BEING ASKED TO PARTICIPATE IN A STUDY ABOUT INFERTILITY

The purpose with this project is to acquire greater knowledge about which lifestyle changes have been made by infertile couples to improve their chances of becoming pregnant and the woman's attitude to sexuality, her physical health and well-being at the start of treatment and on completion of treatment either when she has become pregnant or there are no further options that can be offered. The intention of this study is to increase learn about the need for advice to sub-fertile couples with different cultural and socio-economic backgrounds. The aim is to bridge the gaps in knowledge that are apparent concerning pregnancy planning and life-style changes in relation to infertility and women's pregnancy. The results of the study may be used to develop evidence based advice concerning life-style factors for couples who attend fertility clinics, hopefully leading to improved results after treatment.

We need your contact details to be able to follow up on how you feel and the success of the treatment after approximately two years. The follow-up questions are intended to complement the first questionnaire about changes and influence on life-style during and after the period of treatment.

Your Swedish id-number will also be used to link information to the Swedish birth and pharmaceutical register. This is simply to be able to access information on the results of treatment, possible complications and pregnancy outcomes. Information may also be collected from medical journals about which treatments you have been through and their results. Your details will not be used for any other purpose than for this research project. The results will be analysed at group level and no single individual will be identifiable. Your answers will not be able to lead back to you. The authorities in charge of the study are the Department of Public Health and Caring Sciences (IFV) at Uppsala University and the County Council in Västmanland.

Your contact details will be saved with an encrypted key and will be handled in accordance with the Swedish Personal Data Act (PuL). This means that your personal details will be handled in such a way as to protect your personal integrity. All the material collected: the encrypted key, questionnaires and computer files will be handled only by the project group members and they will be kept in locked places at the Department of public health and caring sciences (IFV)

Participation in the study is voluntary. By filling in your Swedish id-number below you will be agreeing to participate in the project. Do not hesitate to ring or mail us if you have questions using our contact details below. Your consent form will be kept separate from your questionnaire. Your questionnaire will simply have an encrypted key.

Those in charge and contact people for the study are:

Tanja Tydén – Qualified midwife, PhD, Professor at the Department of Public Health and Caring Sciences (IFV), Uppsala University
Tel. 018-471 66 46 • Email: tanja.tyden@pubcare.uu.se

Kjell Wånggren – MD., PhD associate professor, specialist in Obstetrics and Gynaecology, Department of Clinical Science, Intervention and Technology, Karolinska Institutet.
Tel: 08-585 89 752 Email: kjell.wanggren@karolinska.se

Anna Berglund – Head Physician, MD, PhD specialist in Obstetrics and Gynaecology, National Centre for Knowledge on Men's Violence Against Women, Uppsala University.
Tel. 018-611 00 00 • Email: anna.berglund@kbh.uu.se

Lana Salih – Physician, MD. Obstetrics and Gynaecology, Reproduction Centre, Obstetrics and Gynaecology, Uppsala University Hospital
Tel. 018-611 00 00. • Email: lana.salih@kbh.uu.se

UPPSALA UNIVERSITET

Consent Form

I consent to participating in the study of lifestyle, health and infertility. I have been informed about the study in writing and have had enough time to consider my participation. I understand that I am free to conclude my participation in this study at any time.

I consent to my personal details being dealt with as explained in the patient information and I am aware that my participation is completely voluntary.

I will receive a copy of the patient information and this consent form.

I will receive an SF cinema ticket in gratitude of my participation

Swedish ID-number: ☐☐☐☐☐☐-☐☐☐☐

Name:……………………………………………………………………………………………

Address:…………………………………………………………………………………………

…………………………………………………………………………………………………

Mobile no.…………………………………… Tel. no………………………….

Email: ……………………………………………………………………………………………

I prefer to be contacted/receive reminders by:

☐ Text message ☐ Email ☐ Post ☐ Telephone

NB: This page will be separated from the questionnaire and will be stored separately.

UPPSALA
UNIVERSITET

GENERAL QUESTIONS

1. Today's date: Year:……………….. Month:……………….. Day:………………..

2. How old are you? ……………….. years old

3. What is your present occupation/s? (*Tick all relevant options*)

	1-24%	25%	26-49%	50%	51-74%	75%	76-99%	100%
☐ Permanent employment	☐	☐	☐	☐	☐	☐	☐	☐
☐ Self-employed (own business)	☐	☐	☐	☐	☐	☐	☐	☐
☐ Temporary employment	☐	☐	☐	☐	☐	☐	☐	☐
☐ Student	☐	☐	☐	☐	☐	☐	☐	☐
☐ Maternity leave	☐	☐	☐	☐	☐	☐	☐	☐
☐ Sick leave	☐	☐	☐	☐	☐	☐	☐	☐
☐ Unemployed	☐	☐	☐	☐	☐	☐	☐	☐
☐ Housewife/housekeeper	☐	☐	☐	☐	☐	☐	☐	☐
☐ Early retirement due to disease	☐	☐	☐	☐	☐	☐	☐	☐

4. What is the **highest level** of education you have **completed**?

☐ I have not completed any education

☐ Elementary school

☐ Upper secondary school

☐ Vocational school or occupational training

☐ University – 2.5 years or less

☐ University 2.5 - 3.5 years

☐ University – Postgraduate studies

5. What is the monthly income in your household before tax?
(Income is here used to mean: salary, unemployment pay, sickness or parental pay, pension, social benefits, child benefits, widow's benefits, social security benefits and other remunerations.)

…………………. SEK / month

6. How tall are you? ………………..cm

7. How much do you weigh today? ………………..kg

8. How much did you weigh **a year ago?** ………………..kg

9. a. Where were you born? ☐ Sweden ☐ Another country:……………………………………

b. If you were born in a country other than Sweden, how old were you when you came to Sweden? …………years ☐ Less than 1 year old

c. Were you adopted? ☐ Yes ☐ No

10. In which country were your parents born?

a. My mother was born in: ☐ Sweden ☐ Another country: …………………………………………

☐ Don't know

b. My father was born in: ☐ Sweden ☐ Another country: …………………………………………

☐ Don't know

11. How well can you read and understand written Swedish?

Very well	Quite well	Neither well nor badly	Quite badly	Very badly
☐	☐	☐	☐	☐

12. How important is religion in your life?

Very important	Quite important	Neither important nor unimportant	Not very important	Not important at all
☐	☐	☐	☐	☐

SOME QUESTIONS ABOUT YOUR PRESENT PARTNER

13. Which sex is your partner? ☐ Male ☐ Female

14. When was your partner born? Year: 19 …………..

15. How long have you and your partner been in a relationship? (*Tick the option that is nearest.*)

Less than 1 year	1 year	2 years	3 years	4 years	5 years	6 years	7 years	8 years or more
☐	☐	☐	☐	☐	☐	☐	☐	☐

16. What is your relationship with your present partner? *(Tick the best option)*

☐ We have a relationship but don't live together
☐ We are engaged
☐ We live together
☐ We are married

17. a. Has **your present partner** any biological children who are not your biological children?

☐ No ☐ Yes, 1 child ☐ Yes, 2 children ☐ Yes, 3 children ☐ 4 or more children

b. Have **you** any biological children of your own, for whom your present partner is not the biological father?

☐ No ☐ Yes, 1 child ☐ Yes, 2 children ☐ Yes, 3 children ☐ Yes, 4 children or more

18. In which country was your partner born? ☐ Sweden ☐ Another country: …………………………….

☐ Don't know

19. What is the **highest level** of education your partner has **completed**?

☐ Has not completed any education
☐ Elementary and secondary school
☐ Upper secondary school
☐ Vocational school or occupational training
☐ University – 2.5 years or less
☐ University 2.5 - 3.5 years
☐ University – Postgraduate studies

20. Does your present partner smoke? ☐ No ☐ Yes, daily ☐ Yes, but not every day

21. For the following seven questions, tick the answer which <u>most closely</u> describes how you perceive your present relationship.

a. How well does your partner meet your needs in general?	Not at all ☐	☐	Well ☐	☐	Completely ☐
b. How content are you in general with your relationship to your partner?	Not at all ☐	☐	Well ☐	☐	Completely ☐
c. How good is your relationship to your partner in general if you compare it to most other couples?	Much worse ☐	☐	Good ☐	☐	Much better ☐
d. How often do you wish you were not together with your partner?	Never ☐	☐	Sometimes ☐	☐	Always ☐
e. To what extent does your relationship with your partner live up to your original expectations?	Not at all ☐	☐	Well ☐	☐	Completely ☐
f. How much do you like your partner?	Very little ☐	☐	Well enough ☐	☐	Very much ☐
g. How many problems do you have in your relationship?	Very few ☐	☐	Moderate ☐	☐	Very many ☐

YOUR LIFESTYLE JUST NOW

22. What type of food do you eat **just now**? *(Tick the option which* ***best*** *describes your diet)*

- ☐ An all-round diet (You eat most things and do not avoid any specific type of food)
- ☐ Vegetarian food
- ☐ Vegan food
- ☐ I avoid certain foodstuffs because of food hypersensitivity
- ☐ I avoid certain foodstuffs for religious reasons
- ☐ LCHF-diet (Low carbohydrate, high fat diet)
- ☐ GI-food (You eat food with a low GI i.e. glycemic index)
- ☐ Other diet, describe

23. a. Do you take folic acid supplements **just now?**

☐ Yes ☐ No → *Go to question 24*

b. If you take folic acid supplements **just now, when** did you begin taking them?

0-2 weeks ago	3-4 weeks ago	1-2 months ago	3-4 months ago	5-6 months ago	7-12 months ago	1-2 years ago	3-4 years ago	More than 4 years ago
☐	☐	☐	☐	☐	☐	☐	☐	☐

c. **How often** have you taken folic acid supplements during this period?

At least 5 times/week	3 - 4 times/week	1 - 2 times/week	2 - 3 times/month	1 time/month	1 - 6 times/year
☐	☐	☐	☐	☐	☐

24. a. Do you take multivitamin supplements for women **just now?**

☐ Yes ☐ No → *Go to question 25*

b. If you are taking multivitamin supplements for women **just now, when** did you begin to take them?
(Answer in weeks, months, ***or*** *years.)*

0-2 weeks ago	3-4 weeks ago	1-2 months ago	3-4 months ago	5-6 months ago	7-12 months ago	1-2 years ago	3-4 years ago	More than 4 years ago
☐	☐	☐	☐	☐	☐	☐	☐	☐

c. **How often** have you taken the multivitamin supplements for women during this period?

At least 5 times/week	3 - 4 times/week	1 - 2 times/week	2 - 3 times/month	1 time/month	1 - 6 times/year
☐	☐	☐	☐	☐	☐

25. Do you use any pharmaceutical products or naturopathic drugs below? *(Tick all relevant options)*

☐ Nose drops

☐ Medicines for colds

☐ Diuretics

☐ Medicine for pain or inflammation relief

☐ Relaxants

☐ Sleeping pills

☐ Medicines for allergies

☐ Anti-smoking products (e.g. nicotine chewing gum)

☐ Other prescription free drugs

☐ Other naturopathic drugs

☐ No, I only use prescription drugs

☐ No, I use neither medicines nor naturopathic drugs

26. How much coffee do you drink on average a day*(1cup=1.5dl)*? (*Answer in number of cups a day)*

Less than 1 cup a day	1 cup/d.	2 cups/d.	3 cups/d.	4 cups/d.	5 cups/d.	6 cups/d.	7 cups/d.	8 or more cups/d.
☐	☐	☐	☐	☐	☐	☐	☐	☐

or ☐ I don't drink coffee

The following questions are about your alcohol consumption. In these questions we use the term "standard glass of alcohol". A STANDARD GLASS OF ALCOHOL is defined as follows:

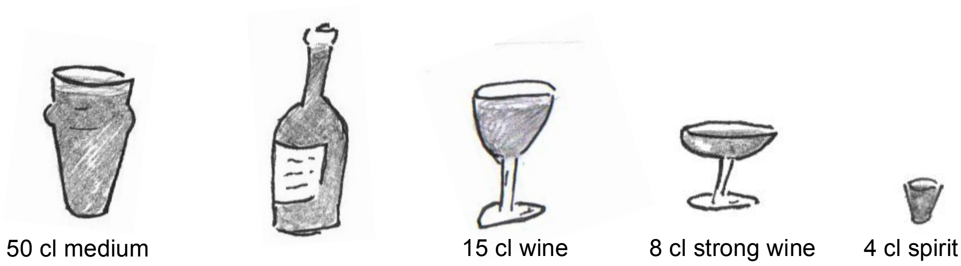

50 cl medium strong beer

33 cl strong beer/ strong cider

15 cl wine

8 cl strong wine

4 cl spirit

27. a. Do you drink alcohol **at present**? *(Tick the option that describes your habits **best**)*

☐ Yes, I drink every week.

☐ Yes, but not every week.

☐ No, I stopped drinking alcohol **when** I started trying to get pregnant this time

☐ No, I had stopped drinking alcohol **before** I started trying to get pregnant this time

☐ No, I have never drunk alcohol

b. How many standard glasses of alcohol a week do you drink on average ***just now?***

Less than 1 glass/w.	1 glass/w.	2 glasses/w.	3 glasses/w.	4 glasses/w.	5 glasses/w.	6 glasses/w.	7 glasses/w.	8 or more glasses/w.
☐	☐	☐	☐	☐	☐	☐	☐	☐

c. How many times during the last **six months** have you drunk **five standard glasses or more at one sitting**?

0 times	1 time	2 times.	3 times.	4 times.	5 times.	6 times.	7 times.	8 or more times
☐	☐	☐	☐	☐	☐	☐	☐	☐

28. a. Do you smoke **just now**? *(Tick the option that **best** describes you)*

- ☐ Yes, I smoke every day.
- ☐ Yes, but not every day.
- ☐ No, I have stopped smoking → *Go to question 29*
- ☐ No, I have never smoked → *Go to question 29*

b. How many cigarettes a day do you smoke on average?

Fewer than 1 cig/day	1 cig/day	2 - 3 cig/day	4 - 5 cig/day	6 - 9 cig/day	10 - 14 cig/day	15 - 20 cig/day	21 - 30 cig/day	31 or more cig/day
☐	☐	☐	☐	☐	☐	☐	☐	☐

29. a. Do you use Swedish snuff tobacco (Sw. 'snus') at present?

- ☐ Yes, every day
- ☐ Yes, but not every day
- ☐ No, I had stopped using it earlier → *Go to question 30*
- ☐ No, I have never used it → *Go to question 30*

b. How many portions of 'snus' a day do you use on average **just now**?

Less than 1 port/day	1 port/day	2 - 3 port/day	4 - 5 port/day	6 - 9 port/day	10 - 14 port/day	15 - 20 port/day	21 - 30 port/day	31 or more port/day
☐	☐	☐	☐	☐	☐	☐	☐	☐

30. Do you use nicotine substitutes *(e.g. nicotine chewing gum or nicotine plasters)* **just now**?

☐ Yes ☐ No

31. How many hours sleep per night have you had on average on a normal weekday this **last month**?

5 or less hrs.	6 hrs.	7 hrs.	8 hrs.	9 hrs.	10 hrs.	11 hrs.	12 hrs.	13 or more hrs.
☐	☐	☐	☐	☐	☐	☐	☐	☐

32. How many hours a week have you spent doing any sort of physical activity this last month? (Round up or down to the nearest hour.)

0 hrs/w	1 hr/w	2 hrs/w	3 hrs/w	4 hrs/w	5 hrs/w	6 - 10 hrs/w	11 – 20 hrs/w	21 or more hrs/w
☐	☐	☐	☐	☐	☐	☐	☐	☐

33. How many times a week on average do you have vaginal intercourse? (Round up or down)

Fewer than 1 time/w.	1 time/w.	2 times./w.	3 times./w.	4 times./w.	5 times./w.	6 times./w.	7 times./w.	8 or more times./w.
☐	☐	☐	☐	☐	☐	☐	☐	☐

34. How satisfied or dissatisfied are you with your sex life?

Very dissatisfied	Quite dissatisfied	Neither satisfied nor dissatisfied	Quite satisfied	Very satisfied
☐	☐	☐	☐	☐

SOME QUESTIONS ABOUT YOUR HEALTH

35. When was the first day of your last period? *(If you can't remember the exact date, please give simply the year and month)*

Year 20……... Month: ……….. Day: ……...

36. How much menstrual pain did you have with your most recent period?

Severe pain	Quite bad pain	Neither severe nor insignificant pain	Mild pain	Hardly any pain
☐	☐	☐	☐	☐

37. How old were you when you had your first menstruation? ……………….. years old

38. How long is your normal **menstrual cycle**? *(A **menstrual cycle** is the time between the first day of one period and the first day of the next)*

20 days or less	21 – 25 days	26 days	27 days	28 days	29 days	30 days	31 – 35 days	36 days or more
☐	☐	☐	☐	☐	☐	☐	☐	☐

39. Do you have, or have you previously been diagnosed with the following disorders/illnesses? *(Tick all relevant options)*

☐ ADHD	☐ Heart condition
☐ Allergy/hypersensitivity to a foodstuff	☐ Hypertension/high blood pressure
☐ Asthma	☐ Chlamydia
☐ Bipolar disorder	☐ Bone pain
☐ Depression	☐ Kidney disorder
☐ Diabetes	☐ Polycystic ovary syndrome (PCOS)
☐ Endometriosis	☐ Back pain
☐ Epilepsy	☐ Anxiety
☐ Cervix Dysplasia (Pap smear)	☐ Eating disorders *(e.g. bulimia or anorexia)*
☐ Thyroid disease *(e.g. Hyper- or hypothyroidism)*	

40. Have you had any illnesses/disorders other than those listed in the previous question?

☐ No ☐ Yes, I have had the following illness(es)/disorder(s): ………………………………………

……

41. Have you ever had an abdominal operation? ☐ Yes ☐ No

42. How is your general health **just now**?

Very good	Quite good	Neither good nor bad	Quite bad	Very bad
☐	☐	☐	☐	☐

PREVIOUS PREGNANCIES

43. a. Have you ever been pregnant *(include miscarriages and abortions)?*

☐ Yes ☐ No → *Go to question 44*

b. How many times have you been pregnant *(include miscarriages and abortions)*?

1 time	2 times.	3 times.	4 times.	5 times.	6 times.	7 times.	8 times.	9 or more times.
☐	☐	☐	☐	☐	☐	☐	☐	☐

c. How old were you when you became pregnant for the first time *(include miscarriages and abortions)*?

……………….. years

d. How many times have you had a miscarriage in months 0-6?

☐ 0 times. ☐ 1 time ☐ 2 times. ☐ 3 times. ☐ 4 times ☐ 5 times. ☐ 6 or more times.

e. How many times have you had an abortion?

☐ 0 times ☐ 1 time ☐ 2 times. ☐ 3 times. ☐ 4 times. ☐ 5 times. ☐ 6 or more times.

f. How many times have you had an ectopic pregnancy?

☐ 0 times ☐ 1 time ☐ 2 times. ☐ 3 times. ☐ 4 times. ☐ 5 times. ☐ 6 or more times.

44. a. Have you ever given birth to a child? ☐ Yes ☐ No → *Go to question 45*

	0	1	2	3	4	5 or more
b. If yes, how many?		☐	☐	☐	☐	☐
c. How many were born alive?	☐	☐	☐	☐	☐	☐
d. How many were born more than 3 months too early?	☐	☐	☐	☐	☐	☐
e. How many were born with a deformation?	☐	☐	☐	☐	☐	☐
f. How many biological children do you have today?	☐	☐	☐	☐	☐	☐

CIRCUMSTANCES AROUND YOUR ON-GOING PREGNANCY ATTEMPT

Below are some questions that concern the circumstances around your on-going attempt to become pregnant. By this we mean <u>the time from</u> when you <u>actively started</u> to try to get pregnant <u>up to and including today's date</u>.

45. Since when have you and your present partner actively tried to get pregnant?

    Years: ……………….. Months: ……………………

46. Who took **the initiative** to the decision that you should try to get pregnant?

    ☐ Just me ☐ Mostly me

    ☐ My partner and I equally

    ☐ Just my partner ☐ Mostly my partner

    ☐ Another person or persons, describe: …………………………………………………

47. Did you decide on a **time plan** within which you expected to get pregnant?

    ☐ Yes, I/we expected that I would get pregnant within …………….. weeks / ……………...month(s)

    ☐ No

48. Have you used an ovulation test during this time that you have been trying to get pregnant?

    ☐ No ☐ Yes, I have used ovulation tests …………… times.

49. During your on-going attempt to get pregnant have you considered adopting a child?

Yes, very often	Yes, a little	Neither yes, nor no	No, not seriously	No, Not at all
☐	☐	☐	☐	☐

50. Have you been given personal advice about infertility or your on-going attempt to get pregnant from any of the following professionals? *(Tick all that are relevant)*

    ☐ Family doctor ☐ Specialist doctor ☐ Midwife

    ☐ Nurse ☐ Dietician ☐ Counsellor

    ☐ Other : ……………………………………………………………………………………………...

    ☐ No, I have not been given any advice about infertility or my on-going attempt to get pregnant

51. Have you, during the time from beginning your on-going attempt to get pregnant, taken any steps to improve your health as a preparation for getting pregnant? *(Tick all that are relevant)*

- ☐ Taken folic acid
- ☐ Stopped smoking ☐ Reduced my smoking
- ☐ Stopped using Swedish snuff tobacco (snus) ☐ Reduced my use of ’snus’
- ☐ Stopped drinking alcohol ☐ Reduced my alcohol consumption
- ☐ Stopped drinking coffee ☐ Reduced my coffee consumption
- ☐ Done more exercise ☐ Done less exercise
- ☐ Changed your medications
- ☐ Eaten more healthily, describe: ………………………………………………………………………………
- ☐ Sought medical/health advice
- ☐ Made other choices, describe: ………………………………………………………………………

or

- ☐ I have not done any of the above

52. Where have you and/or your partner sought information about infertility or your on-going attempt to get pregnant? *(Tick all that are relevant)*

- ☐ Maternity / Midwife Clinic
- ☐ Other health clinic
- ☐ Family, friends and acquaintances
- ☐ Livsmedelsverket (Sweden’s National Food Agency)
- ☐ Folkhälsomyndigheten (Public Health Agency of Sweden)
- ☐ Internet forums *(internet sites for: pregnancy, family life, growing people, parents)*
- ☐ Health sites on internet *(e.g. 1177 Vårdguiden)*
- ☐ Other internet sites:…………………………………………………………………………………………...
- ☐ Newspapers/magazines
- ☐ Books
- ☐ Other: ……..
- ☐ No, I have not sought information about infertility or my on-going attempt to get pregnant

53. Tick the answer which best describes how you have been **this week**.

a. I have felt tense and nervous	Very often ☐	Often ☐	Rarely ☐	Not at all ☐
b. I have a feeling something terrible is going to happen	Very strongly and unpleasantly ☐	Not so strongly just now ☐	Much less just now ☐	Not at all ☐
c. I worry about things	Very often ☐	Often ☐	Rarely ☐	Not at all ☐
d. I can sit still and feel relaxed	Very often ☐	Often ☐	Rarely ☐	Not at all ☐
e. I feel worried, as if I had butterflies in my stomach	Very often ☐	Often ☐	Rarely ☐	Not at all ☐
f. I feel restless	Very often ☐	Often ☐	Rarely ☐	Not at all ☐
g. I get sudden panic attacks	Very often ☐	Often ☐	Rarely ☐	Not at all ☐

54. For the next 10 questions, tick the option which best describes how you have been feeling **this week**.

a. I have been able to laugh and see the bright side of life<br><br>☐ As normal<br>☐ Nearly as good as normal<br>☐ Much worse than normal<br>☐ Not at all	f. Things have been getting on top of me<br><br>☐ Yes, most of the time I haven't been able to do anything at all<br>☐ Yes, sometimes I haven't been able to cope as well as usual<br>☐ No, mostly I have managed to cope quite well<br>☐ No, I've been able to do things as usual
b. I've been happy about things that have happened<br><br>☐ As much as normal<br>☐ Less than normal<br>☐ Much less than normal<br>☐ Not at all	g. I've felt so unhappy that I've had problems sleeping<br><br>☐ Yes, very much so<br>☐ Yes, sometimes<br>☐ No, rarely<br>☐ No, never
c. I've blamed myself too much when things have gone wrong<br><br>☐ Yes, most of the time<br>☐ Yes, sometimes<br>☐ Not often<br>☐ No, never	h. I have felt sad and miserable<br><br>☐ Yes, most of the time<br>☐ Yes, quite often<br>☐ No, rarely<br>☐ No, never
d. I have felt frightened and worried without any special reason<br><br>☐ No, Not at all<br>☐ Quite rarely<br>☐ Yes, sometimes<br>☐ Yes, very often	i. I've felt unhappy and cried<br><br>☐ Yes, most of the time<br>☐ Yes, quite often<br>☐ Only once or twice<br>☐ Never
e. I have felt frightened and full of panic without any special reason<br><br>☐ Yes, very often<br>☐ Yes, sometimes<br>☐ No, quite rarely<br>☐ No, Not at all	j. I've had thoughts about hurting myself<br><br>☐ Yes, very often<br>☐ Yes, now and again<br>☐ Rarely<br>☐ Never

55.During **the last month** how often have you …

		Never	Rarely	Some-times	Quite often	Very often
a.	… been upset about something unexpected happening?	☐	☐	☐	☐	☐
b.	… felt you haven’t had control over important issues in your life?	☐	☐	☐	☐	☐
c.	… felt nervous and under stress?	☐	☐	☐	☐	☐
d.	… felt capable of coping with personal problems?	☐	☐	☐	☐	☐
e.	… felt that things have turned out as you wanted?	☐	☐	☐	☐	☐
f.	… felt that you could deal with everything that has been needing to be done?	☐	☐	☐	☐	☐
g.	… felt you could cope with irritating moments in your life?	☐	☐	☐	☐	☐
h.	… felt that you have been in control of things?	☐	☐	☐	☐	☐
i.	… felt angry about things that have happened and that have been outside your control?	☐	☐	☐	☐	☐
j.	… felt that there have been so many problems that you haven’t been able to cope?	☐	☐	☐	☐	☐

PREGNANCY IN GENERAL

These questions are about how you feel about your ability to become pregnant. NB This is not a test of knowledge – we just want to know what you feel.

56. What do you think is the average chance for a woman of your age to become pregnant if she has unprotected vaginal intercourse **while she is ovulating**?

    .... percent chance at the time of ovulation

57. What do you think is the average chance, at each attempt, for a woman of your age to become pregnant **with the help of test tube fertilisation**?

    .... percent chance at each attempt

58. At which age do you think women are most fertile? .... years

59. At what age do you think that a woman's chance to become pregnant starts to decline **to some extent**? .... years

60. At what age do you think that a woman's chance to become pregnant **clearly** starts declining? .... years

61. Have you experienced the following as supportive or hindering you having children?

	Very supportive	Quite supportive	Neither supportive nor hindering	Quite hindering	Very hindering	Not relevant
Your partner	☐	☐	☐	☐	☐	☐
Your family	☐	☐	☐	☐	☐	☐
Your friends	☐	☐	☐	☐	☐	☐
Your age	☐	☐	☐	☐	☐	☐
Your maturity	☐	☐	☐	☐	☐	☐
Your housing situation	☐	☐	☐	☐	☐	☐
Your career	☐	☐	☐	☐	☐	☐
Your employment situation	☐	☐	☐	☐	☐	☐
Your finances	☐	☐	☐	☐	☐	☐
Which paternity benefits you are entitled to	☐	☐	☐	☐	☐	☐

SOME QUESTIONS ABOUT YOUR UPBRINGING

These questions are about your childhood. If the questions raise a need for you to talk to an outsider, you can ask your doctor who will show you how to find the help you need.

62. During the first 18 years of your life:

	Yes	No
a. Did you live together with someone who had problems with alcohol consumption, or was indeed an alcoholic?	☐	☐
b. Did you live together with someone who used drugs?	☐	☐
c. Did your parents ever separate or divorce?	☐	☐
d. Was there anybody in your home who was depressed or mentally ill?	☐	☐
e. Was there anybody in your home who tried to commit suicide	☐	☐
f. Was there anybody in your home who was ever imprisoned?	☐	☐

63. During the first 18 years of your life:

	Never	Once or twice	Some-times	Often	Very often
a. How often did you feel that nobody in your family loved you or thought that you were important or special?	☐	☐	☐	☐	☐
b. How often did you feel that people in your family didn't care about each other, weren't emotionally close and didn't support each other?	☐	☐	☐	☐	☐
c. How often did you have too little to eat, had to wear dirty clothes and felt that nobody took care of you?	☐	☐	☐	☐	☐
d. How often were your parents too inebriated with drink or drugs to look after you or take you to the doctor if necessary?	☐	☐	☐	☐	☐

64. Sometimes parents or other adults hurt children. During your childhood, i.e. the first 18 years of your life, how often did a parent, a step-parent or other adult living in your home:

	Never	Once or twice	Sometimes	Often	Very often
a. Swear at you, insult you or violate you?	☐	☐	☐	☐	☐
b. Threatened to hit you or throw something at you, but never did?	☐	☐	☐	☐	☐
c. Reacte in such a way that made you scared that you might be hurt physically?	☐	☐	☐	☐	☐
d. Actually hit you, grab you, push you or throw something at you?	☐	☐	☐	☐	☐
e. Hit you so hard that it left marks or injured you?	☐	☐	☐	☐	☐

65. Sometimes parents are physically violent with each other. During the first 18 years of your life how often did your father (or step-father) or your mother's boyfriend/partner do any of the following **things to your mother (or stepmother)**?

	Never	Once or twice	Sometimes	Often	Very often
a. Pushed, grabbed, hit or threw something at her?	☐	☐	☐	☐	☐
b. Kicked, bit or hit her with his fists or something hard?	☐	☐	☐	☐	☐
c. Hit her repeatedly for at least several minutes?	☐	☐	☐	☐	☐
d. Threatened her with a knife or gun with the intention of hurting her?	☐	☐	☐	☐	☐

66. Sometimes parents are physically violent with each other. During the first 18 years of your life how often did your mother (or stepmother) or your father's girlfriend/partner do any of these **things to your father (or stepfather)**?

	Never	Once or twice	Sometimes	Often	Very often
a. Pushed, grabbed, hit or threw something at him?	☐	☐	☐	☐	☐
b. Kicked, bit or hit him with her fists or something hard?	☐	☐	☐	☐	☐
c. Hit him repeatedly for at least several minutes?	☐	☐	☐	☐	☐
d. Threatened him with a knife or gun with the intention of hurting him?	☐	☐	☐	☐	☐

67. Some people, during the first 18 years of their life, have had sexual experiences with an adult or somebody at least 5 years older than themselves. These experiences may have involved a relative, family friend or a stranger. During the first 18 years of your life did any adult or older relative, family friend or stranger:

	How old were you the first time this happened?	The first time it happened, was it against your will?	How old were you the last time this happened?	Roughly how many times did it happen to you?	How many people did this to you?	What sex were those who did it?
a. Touch or caress your body in a sexual way? ☐ Yes ☐ No If "Yes" →	---------------- years	☐ Yes ☐ No	---------------- years	---------------- times	-------------- people	☐ male ☐ female ☐ both
b. Make you touch or caress their body in a sexual way? ☐ Yes ☐ No If "Yes" →	---------------- years	☐ Yes ☐ No	---------------- years	---------------- times	-------------- people	☐ male ☐ female ☐ both
c. Try to have some kind of sexual intercourse (oral, anal or vaginal) with you? ☐ Yes ☐ No If "Yes" →	---------------- years	☐ Yes ☐ No	---------------- years	---------------- times	-------------- people	☐ male ☐ female ☐ both
d. Succeed in having sexual intercourse (oral, anal or vaginal) with you? ☐ Yes ☐ No If "Yes" →	---------------- years	☐ Yes ☐ No	---------------- years	---------------- times	-------------- people	☐ male ☐ female ☐ both

68. Did you find the questions about your childhood (questions 62-67) unpleasant to answer?

☐ Yes, I don't want to answer

☐ Yes, but it feels alright to answer

☐ No, it was alright to answer them

☐ Don't know/Have no opinion

ABOUT YOUR VISIT TO OUR CLINIC

69. What expectations do you have for your visit at our clinic?

☐ To know why I have not become pregnant, and to get treatment as quickly as possible

☐ To know why I have not become pregnant, but I want to postpone any treatment and carry on trying on my own a little longer

☐ Other, describe: ……………………………………………………………………………………………

………..

………..

………..

………..

………..

………..

………..

………..

………..

………..

………..

SOME LAST QUESTIONS

70. Has your partner been with you while you have been filling in this questionnaire?

☐ Yes ☐ No

71. Has anybody helped you fill in this questionnaire?

☐ No, nobody has helped me

☐ Yes, my husband/partner

☐ Yes, an interpreter

☐ Yes, the doctor/the nurse

☐ Yes, somebody else. Who?: …………………………………………………………………...

COMMENTS ABOUT THE QUESTIONNAIRE

If you wish to make any additions to, or comments about this questionnaire, e.g. if any question was unclearly put, if you want to add something to any question, or if you thought there was a question that we missed, you may do so here. We appreciate all your comments.

……

……

……

……

……

……

……

……

……

……

……

……

……

……

……

……

……

……

……

……

……

THANK YOU FOR YOUR HELP

If this questionnaire has raised a need for you to talk to an outsider, you can ask your doctor who will show you how to find the help you need.

Encrypted key:………………..
